# Burkholderia ubonensis Meropenem Resistance: Insights into Distinct Properties of Class A β-Lactamases in Burkholderia cepacia Complex and Burkholderia pseudomallei Complex Bacteria

**DOI:** 10.1128/mBio.00592-20

**Published:** 2020-04-14

**Authors:** Nawarat Somprasong, Carina M. Hall, Jessica R. Webb, Jason W. Sahl, David M. Wagner, Paul Keim, Bart J. Currie, Herbert P. Schweizer

**Affiliations:** a Department of Molecular Genetics & Microbiology, College of Medicine, Emerging Pathogens Institute, University of Florida, Gainesville, Florida, USA; b The Pathogen and Microbiome Institute, Northern Arizona University, Flagstaff, Arizona, USA; c Global and Tropical Heath Division, Menzies School of Health Research, Darwin, Northern Territory, Australia; d Department of Infectious Diseases, Royal Darwin Hospital, Darwin, Northern Territory, Australia; e Northern Territory Medical Program, Royal Darwin Hospital, Darwin, Northern Territory, Australia; McMaster University

**Keywords:** *Burkholderia*, antibiotics, resistance, efflux pump, adaptation, β-lactamases, β-lactams, induction, meropenem

## Abstract

Burkholderia pseudomallei, the founding member of the B. pseudomallei complex (Bpc), is a biothreat agent and causes melioidosis, a disease whose treatment mainly relies on ceftazidime and meropenem. The concern is that B. pseudomallei could enhance its drug resistance repertoire by the acquisition of DNA from resistant near-neighbor species. Burkholderia ubonensis, a member of the B. cepacia complex (Bcc), is commonly coisolated from environments where B. pseudomallei is present. Unlike B. pseudomallei, in which significant primary carbapenem resistance is rare, it is not uncommon in B. ubonensis, but the underlying mechanisms are unknown. We established that carbapenem resistance in B. ubonensis is due to an inducible class A PenB β-lactamase, as has been shown for other Bcc bacteria. Inducibility is not sufficient for high-level resistance but also requires other determinants, such as a PenB that is more robust than that present in susceptible isolates, as well as other resistance factors. Curiously and diagnostic for the two complexes, both Bpc and Bcc bacteria contain distinct annotated PenA class A β-lactamases. However, the protein from Bcc bacteria is missing its essential active-site serine and, therefore, is not a β-lactamase. Regulated expression of a transcriptional *penB*′-*lacZ* (β-galactosidase) fusion in the B. pseudomallei surrogate B. thailandensis confirms that although Bpc bacteria lack an inducible β-lactamase, they contain the components required for responding to aberrant peptidoglycan synthesis resulting from β-lactam challenge. Understanding the diversity of antimicrobial resistance in *Burkholderia* species is informative about how the challenges arising from potential resistance transfer between them can be met.

## Abstract (precis)

Burkholderia pseudomallei causes melioidosis, a tropical disease that is highly fatal if not properly treated. Our data show that, in contrast to B. pseudomallei, B. ubonensis β-lactam resistance is fundamentally different because intrinsic resistance is mediated by an inducible class A β-lactamase. This includes resistance to carbapenems. Our work demonstrates that studies with near-neighbor species are informative about the diversity of antimicrobial resistance in *Burkholderia* and can also provide clues about the potential of resistance transfer between bacteria inhabiting the same environment. Knowledge about potential adverse challenges resulting from the horizontal transfer of resistance genes between members of the two complexes enables the design of effective countermeasures.

## INTRODUCTION

Burkholderia pseudomallei is a Gram-negative bacterium inhabiting water and soil in regions of endemicity that span the tropical and subtropical regions of the globe (1, 2). The bacterium is the founding member of the B. pseudomallei complex (Bpc) (3) and is considered a biothreat agent. B. pseudomallei infections are difficult to treat due to intrinsic antibiotic resistance, which limits therapy to just a few antibiotics (4–6). Primary infection isolates that are collected from melioidosis patients prior to antibiotic exposure are naturally susceptible to the expanded-spectrum β-lactam antibiotics (e.g., ceftazidime [CAZ] and carbapenems [meropenem {MEM} and imipenem {IMP}]) used for melioidosis treatment (2, 6). Acute-phase melioidosis therapy consists of CAZ and/or MEM, which is critical for a successful clinical outcome, and even though acquired resistance to these antibiotics during therapy is uncommon, at least CAZ resistance (CAZ^r^) has been increasingly detected (7). Although deletion of penicillin-binding protein 3 (PBP 3) has been documented as a clinically significant CAZ^r^ mechanism (8), mutations causing class A PenA β-lactamase overexpression (promoter upmutation, creating a stronger promoter, and gene duplication and amplification) and amino acid substitutions are the main causes of acquired CAZ^r^ in B. pseudomallei (9–15). Environmental isolates as well as primary isolates taken from melioidosis patients are susceptible to MEM (MICs, <1 to 2 μg/ml), and carbapenem resistance is less common than CAZ^r^. However, in rare Australian B. pseudomallei isolates, decreased MEM susceptibility (MICs, 3 to 8 μg/ml) has been noted and has been attributed to (i) mutations affecting PenA expression and changes in critical amino acid residues (14) and (ii) efflux in regulatory mutants affecting AmrAB-OprA or BpeAB-OprB efflux pump expression, arising during MEM therapy (16). PenA is the only active β-lactamase in B. pseudomallei, and its expression is not inducible by β-lactam substrates (17). Even though β-lactam resistance and resistance to other antibiotics are rare in B. pseudomallei, possible enhancement of the resistance repertoire by acquisition of DNA from drug-resistant near-neighbor Bpc or B. cepacia complex (Bcc) species is of concern. Although there is no direct evidence for genetic transfer between Bpc and Bcc species, about half of all B. pseudomallei strains are naturally competent for non-source-specific DNA uptake, which could facilitate horizontal gene transfer in environments that the bacteria coinhabit (18, 19).

Burkholderia ubonensis is a Gram-negative Bcc bacterium that can be commonly isolated from water and soil (20, 21). Along with other Bcc members, this bacterium is regularly coisolated from the environment with B. pseudomallei and is considered nonpathogenic. For unknown reasons, high-level antibiotic resistance is more common in B. ubonensis than in other Bcc bacteria. In contrast to other Bcc bacteria and B. pseudomallei, MEM resistance (MEM^r^) is not uncommon in B. ubonensis, but the β-lactam resistance mechanisms in this bacterium have not yet been elucidated (21). High-level (≥32 μg/ml) MEM^r^ is frequent in isolates from Puerto Rico but is less frequently observed in Australian isolates, where MEM MIC levels range from ≥32 μg/ml to 2 μg/ml (21).

**FIG 1:**
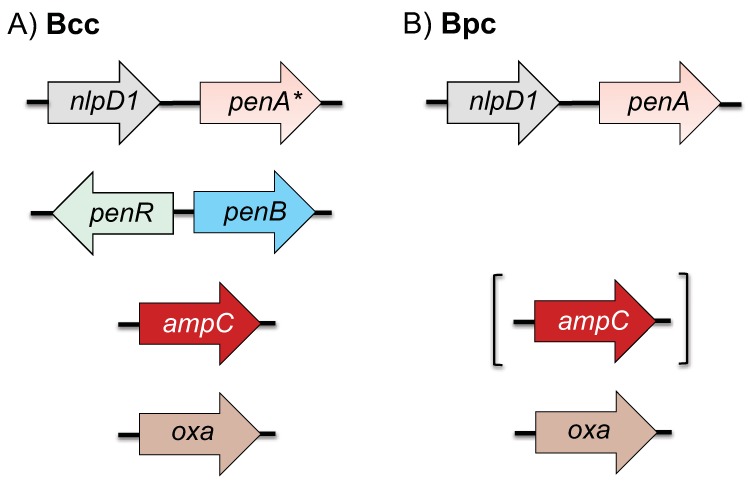
Genetic organization of annotated β-lactamase-encoding genes in B. cepacia complex (Bcc) (A) and B. pseudomallei complex (Bpc) bacteria (B). The *penA* and *penB* genes encode the PenA and PenB class A β-lactamases, respectively, which are genetically localized and expressed in a defined context in Bcc and Bpc bacteria (see Fig. S1 in the supplemental material). PenA confers β-lactam resistance in Bpc bacteria. Bcc bacteria express PenA proteins (PenA*) that do not exhibit β-lactamase activity. In Bcc bacteria, PenB (previously annotated with species-specific names) confers β-lactam resistance. AmpC is a class C β-lactamase. Class C β-lactamases are absent from significant Bpc bacteria, such as B. mallei, B. pseudomallei, and B. thailandensis, but proteins with homology to AmpCs from other bacteria are present in few other Bpc bacteria (indicated by brackets). Class D β-lactamases are annotated as OXA enzymes in Bcc and Bpc bacteria, but the evidence for their biological activities is weak and evidence with respect to clinical significance is lacking. PenR is a LysR-type regulator which governs the expression of PenB and AmpC in Bcc bacteria in response to β-lactam challenge. In B. pseudomallei, the *penA* gene was shown to reside in the same operon as *nlpD1* (Fig. S1). *nlpD1* encodes a membrane-bound lipoprotein with peptidase and peptidoglycan-binding domains; NlpD1 may function as an activator of lytic cell wall amidase activity. There is currently no published evidence for the inducibility of *penA* and penA* expression.

Currently, there are four molecular classes of β-lactamases, classes A, B, C, and D (22). Class A, C, and D enzymes contain an active-site serine, and class B enzymes are Zn^2+^ metalloenzymes (22). Bcc and Bpc bacteria contain chromosomally encoded representatives of class A, C, and D β-lactamases (Fig. 1). Bcc bacteria encode two annotated class A β-lactamases, PenA* (an inactive homolog of Bpc PenA, first noted in B. multivorans [23]) and PenB, as well as the class C enzyme AmpC (Fig. 1) (24). To avoid widespread confusion caused by assigning a different name to the same enzymes in Bpc and Bcc bacteria (25), we employ a uniform nomenclature for the proteins found in the respective bacteria. For Bpc bacteria, we use PenA, which has traditionally been used to describe the lone class A β-lactamase in B. pseudomallei and the closely related species B. mallei (9). For Bcc bacteria, we use PenB, whose sequence was first described in B. cenocepacia (25). The genes for PenA, PenA*, and PenB are genetically localized in a defined context in Bcc and Bpc bacteria (15) (see Fig. S1 in the supplemental material). Like B. pseudomallei, the B. ubonensis genome encodes a putative class D OXA-like serine β-lactamase (Fig. 1), and some of these enzymes exhibit carbapenemase activity in diverse Gram-negative pathogens. However, this enzyme has not been demonstrated to confer β-lactam/carbapenem resistance in clinical or environmental B. pseudomallei isolates.

10.1128/mBio.00592-20.2 FIG S1 Genomic organization of *penA*, penA*, and *penB* loci in *Burkholderia* species. Download FIG S1, PDF file, 0.2 MB. Copyright © 2020 Somprasong et al. 2020 Somprasong et al. This content is distributed under the terms of the Creative Commons Attribution 4.0 International license.

In many Gram-negative bacteria, including Bcc bacteria, a β-lactamase(s) is induced in response to peptidoglycan perturbation, which in turn results from transpeptidase inhibition by β-lactam antibiotics. In *Enterobacteriaceae* and Pseudomonas aeruginosa, this regulation is mediated by the AmpR/AmpC regulatory pathway (26, 27), and the analogous system in B. cenocepacia is PenR/PenB (24, 28). PenR is a LysR-type protein encoded by a gene that is transcribed divergently from *penB* (Fig. 1). AmpR-type proteins are bifunctional transcriptional regulators, functioning either as a repressor or as an activator. Binding of the cell wall precursor UDP-MurNAc-pentapeptide to PenR leads to repression of *penB* and *ampC* transcription. According to the model (26, 27), PenR functions as an activator after binding of either GlcNAc-1,6-anhydroMurNAc-pentapeptide or 1,6-anhydroMurNAc-pentapeptide. The first is the product of periplasmic soluble lytic transglycosylase (Slt), which cleaves the accumulated nascent non-cross-linked peptidoglycan that accumulates as a result of β-lactam inhibition of the transpeptidase domain of bifunctional high-molecular-weight penicillin-binding proteins (PBPs). The liberated GlcNAc-1,6-anhydroMurNAc-pentapeptide is transported into the cell by the AmpG permease. Cytoplasmic GlcNAc-1,6-anhydroMurNAc-pentapeptide either binds PenR for PenB and AmpC β-lactamase activation or is cleaved by β-*N*-acetylhexosaminidase (NagZ). The resulting 1,6-anhydroMurNAc-pentapeptide also functions to activate PenR.

The main goals of this study were to elucidate the mechanism(s) underpinning the high-level MEM^r^ (MIC, ≥32 μg/ml) in B. ubonensis strain Bu278 (also known as Bp8955) by comparison to a susceptible strain and to assess whether Bpc bacteria are able to express exogenous β-lactam resistance determinants in the same manner as B. ubonensis after acquiring its resistance genes, possibly via horizontal gene transfer, for which there is ample evidence in B. pseudomallei (29).

## RESULTS

### Identification of B. ubonensis mutants with reduced meropenem susceptibility.

To assess the resistance determinants that form the basis for the high MEM^r^ (MIC, ≥32 μg/ml) of strain Bu278 (the B. ubonensis strains used in this study are listed in Table S1 in the supplemental material), random transposon mutagenesis was performed. A screen of 3,515 transformants identified 10 mutants with increased MEM susceptibility, as defined by no growth on Lennox broth (LB) plates containing 8 μg/ml MEM. The meropenem MICs for all 10 mutants were determined and ranged from 1.5 μg/ml (susceptible) to 24 μg/ml (resistant) (Table S2). With the exception of two insertions in a gene encoding the outer membrane (OM) protein assembly factor BamC, unique insertions were in genes encoding diverse cellular functions. This distribution may be indicative of the diversity of factors governing β-lactam resistance in *Burkholderia*, as previously established for a ceftazidime (CAZ)- and imipenem (IMP)-resistant B. pseudomallei strain (15). Transposon or deletion mutants deficient in soluble lytic transglycosylase (Slt) and β-*N*-acetylhexosaminidase (NagZ) exhibited the lowest MEM MIC (1.5 μg/ml) (Tables 1 to 3; Table S2). The Δ*nagZ* and Δ*slt* mutations were complemented by single-copy expression of the respective wild-type genes from the leaky *P_BAD_* promoter (Table 2). These observations are consistent with these two enzymes playing a crucial role in β-lactamase induction, as has been established with other bacteria, and hinted at MEM^r^ being caused by an inducible carbapenemase.

**TABLE 1:** β-Lactam resistance of B. ubonensis Bu278 and its derivatives

Strain	Relevant genotype	MIC (μg/ml)^*a*^			
		MEM	IMP	CAZ	AMX
Bu278	Wild type	≥32	≥32	8	≥256
Bu290	Bu278 *slt*::T23	1.5	6	1.5	≥256
Bu295	Bu278 *nagZ*::T23	1.5	6	1.5	≥256
Bu296	Bu278 Δ*slt*	1.5	6	1.5	≥256
Bu308	Bu278 Δ*nagZ*	1.5	4	2	≥256
Bu311	Bu278 ΔpenA*	≥32	≥32	8	≥256
Bu312	Bu278 Δ*penB*	0.75	0.19	1.5	192
Bu314	Bu278 Δ*ampC*	≥32	≥32	8	≥256

a The MIC was determined using the Etest method, performed in triplicate on three separate days, and values are reported as the mode of the readings.

**TABLE 2:** β-Lactam resistance of B. ubonensis Bu278 complemented mutants

Strain	Relevant genotype	MIC (μg/ml)^*a*^							
		MEM		IMP		CAZ		AMX	
		−Ara	+Ara	−Ara	+Ara	−Ara	+Ara	−Ara	+Ara
Bu338	Bu278::mini-Tn*7*-*P_BAD_*	≥32	≥32	≥32	≥32	8	12	≥256	≥256
Bu352	Bu308::mini-Tn*7*-*P_BAD_*	2	2	4	4	2	2	≥256	≥256
Bu373	Bu308::mini-Tn*7*-*P_BAD_*- *nagZ^+^*	≥32	≥32	≥32	≥32	4	16	≥256	≥256
Bu356	Bu296::mini-Tn*7*-*P_BAD_*	1.5	2	6	6	1.5	2	≥256	≥256
Bu375	Bu296::mini-Tn*7*-*P_BAD_-slt^+^*	≥32	≥32	≥32	≥32	4	24	≥256	≥256
Bu382	Bu312::mini-Tn*7*	0.75	NA	0.19	NA	2	NA	192	NA
Bu379	Bu312::mini-Tn*7*-*P_penB_*- *penB*_Bu278_*^+^*	≥32	NA	≥32	NA	8	NA	≥256	NA

a The MIC was determined using the Etest method, performed in triplicate on three separate days, and values are reported as the mode of the readings. −Ara, medium without l-arabinose; +Ara, medium with l-arabinose at a final concentration of 1%. NA, not applicable.

**TABLE 3:** Avibactam inhibition of β-lactam resistance in B. ubonensis Bu278^*a*^

MIC (μg/ml)^*b*^							
MEM		IMP		CAZ		AMX	
−AVI	+AVI	−AVI	+AVI	−AVI	+AVI	−AVI	+AVI
32	1	32	0.5	16	2	ND	ND

a Avibactam inhibition was assessed by broth microdilution, performed in triplicate and on two separate days; the avibactam concentration was kept constant at 4 μg/ml.

b MIC values are reported as the mode of the readings. −AVI, medium without avibactam; +AVI, medium without avibactam; ND, not done.

10.1128/mBio.00592-20.4 TABLE S1 Burkholderia ubonensis strains used in this study. Download Table S1, PDF file, 0.1 MB. Copyright © 2020 Somprasong et al. 2020 Somprasong et al. This content is distributed under the terms of the Creative Commons Attribution 4.0 International license.

10.1128/mBio.00592-20.5 TABLE S2 B. ubonensis transposon mutants with increased meropenem susceptibility. Download Table S2, PDF file, 0.1 MB. Copyright © 2020 Somprasong et al. 2020 Somprasong et al. This content is distributed under the terms of the Creative Commons Attribution 4.0 International license.

### β-Lactam antibiotics induce PenB and AmpC, but not PenA* and OXA.

To ascertain the cadre of β-lactamases potentially induced by β-lactam antibiotics that are clinically significant in Bcc and Bpc bacteria, we measured the mRNA levels of penA*, *penB*, *ampC*, and *oxa* in Bu278 cells that were grown to log phase and then either challenged for 1 h with subinhibitory concentrations of 8 μg/ml IMP and MEM or 3 μg/ml CAZ or left uninduced (containing no antibiotics) (Table 4). These concentrations were chosen because they were subinhibitory for Bu278 (Tables 1 to 3). The data show that expression of *penB* and *ampC* was highly inducible by IMP (371-fold and 99-fold, respectively) and MEM (337-fold and 64-fold, respectively) and was inducible to a lesser extent by CAZ (18-fold and 9-fold, respectively). In contrast, penA* and *oxa* expression was not inducible, at least not with the tested β-lactams and under the conditions employed in this study. To test the dependency of β-lactamase induction on Slt and NagZ, we measured penA*, *penB*, and *ampC* mRNA levels in the wild-type strain and its Δ*slt* and Δ*nagZ* derivatives challenged for 1 h with 1 μg/ml of IMP, MEM, or CAZ; these concentrations were chosen since they were subinhibitory for all strains tested in this experiment (Tables 1 to 3) and allowed growth of the Δ*slt* and Δ*nagZ* mutants yet reproducibly induced *penB* and *ampC* in the wild-type strain Bu278, although the induction levels were not as high as those observed with higher inducer concentrations, especially in MEM-challenged cells (Table 5). The data also show that the IMP- and MEM-induced *penB* and *ampC* expression was significantly lower in the Δ*slt* and Δ*nagZ* mutants than in the wild-type Bu278 strain. Although the impact of the deletion of Slt was more severe than that of the deletion of NagZ, *penB* and *ampC* expression was not completely dependent on Slt and NagZ. In contrast, CAZ-induced *penB* and *ampC* expression was nearly abolished in the Δ*slt* and Δ*nagZ* mutants. The effects of the Slt and NagZ deletions were the most pronounced with PenB in IMP-challenged cells, and this was reflected in IMP and MEM resistance levels (Tables 1 to 3; see below). Otherwise, the PenB and AmpC mRNA levels in IMP- and MEM-challenged cells were a poor predictor of the MICs.

**TABLE 4:** β-Lactam challenge-induced β-lactamase expression in B. ubonensis^*a*^

Gene	Treatment							
	Untreated		MEM		IMP		CAZ	
	Normalized fold mRNA expression ± SD	*P* value	Normalized fold mRNA expression ± SD	*P* value	Normalized fold mRNA expression ± SD	*P* value	Normalized fold mRNA expression ± SD	*P* value
penA*	1.00 **±** 0.05	NA	0.79 **±** 0.10	0.98	0.47 **±** 0.03	0.94	0.75 **±** 0.05	0.35
*penB*	1.00 **±** 0.13	NA	336.94 **±** 35.58	<0.001	371.00 **±** 45.40	<0.001	17.65 **±** 1.41	<0.001
*ampC*	1.00 **±** 0.12	NA	64.43 **±** 9.03	<0.001	99.31 **±** 6.81	<0.001	9.09 **±** 0.77	<0.001
*oxa*	1.00 **±** 0.07	NA	0.80 **±** 0.17	0.98	0.86 **±** 0.05	0.99	0.70 **±** 0.07	0.28

a Cells of Bu278 (wild type) were grown to log phase in LB medium. Equal portions of the cell cultures remained untreated or were treated with subinhibitory concentrations of meropenem (MEM; 8 μg/ml), imipenem (IMP; 8 μg/ml), or ceftazidime (CAZ; 3 μg/ml). Total RNA was isolated after an additional 1 h of incubation at 37°C. The penA*, *penB*, *ampC*, and *oxa* mRNA levels were determined by RT-qPCR. The penA*, *penB*, *ampC*, and *oxa* mRNA levels were determined by RT-qPCR. Standard deviations (SD) between three biological replicates are indicated. Two-way ANOVA and Sidak’s multiple-comparison test were used to determine the significance of the change in fold mRNA expression levels between treated (with MEM, IPM, or CAZ) and untreated strains. *P* values of <0.05 were considered significant. NA, not applicable.

**TABLE 5:** β-Lactam challenge-induced β-lactamase expression in Δ*slt* and Δ*nagZ* mutants^*a*^

Treat- ment	Gene	Bu278				Bu278 Δ*slt*				Bu278 Δ*nagZ*			
		Untreated		Treated		Untreated		Treated		Untreated		Treated	
		Normalized fold mRNA expression ± SD	*P* value	Normalized fold mRNA expression ± SD	*P* value	Normalized fold mRNA expression ± SD	*P* value	Normalized fold mRNA expression ± SD	*P* value	Normalized fold mRNA expression ± SD	*P* value	Normalized fold mRNA expression ± SD	*P* value
MEM	penA*	1.00 **±** 0.05	NA	0.90 **±** 0.16	0.96	1.00 **±** 0.11	NA	0.87 **±** 0.08	0.82	1.00 **±** 0.07	NA	0.92 **±** 0.07	0.91
	*penB*	1.00 **±** 0.08	NA	61.33 **±** 11.39	<0.001	1.00 **±** 0.12	NA	22.86 **±** 2.87	<0.001	1.00 **±** 0.11	NA	28.52 **±** 3.19	<0.001
	*ampC*	1.00 **±** 0.20	NA	14.66 **±** 3.56	<0.001	1.00 **±** 0.15	NA	4.99 **±** 0.75	<0.001	1.00 **±** 0.12	NA	21.37 **±** 2.23	<0.001
IMP	penA*	1.00 **±** 0.12	NA	0.94 **±** 0.19	0.99	1.00 **±** 0.10	NA	0.73 **±** 0.09	0.38	1.00 **±** 0.10	NA	0.87 **±** 0.07	0.94
	*penB*	1.00 **±** 0.12	NA	274.45 **±** 51.20	<0.001	1.00 **±** 0.10	NA	13.17 **±** 1.56	<0.001	1.00 **±** 0.15	NA	58.61 **±** 5.77	<0.001
	*ampC*	1.00 **±** 0.07	NA	61.76 **±** 10.27	<0.001	1.00 **±** 0.08	NA	1.95 **±** 0.20	0.003	1.00 **±** 0.14	NA	54.14 **±** 6.19	<0.001
CAZ	penA*	1.00 **±** 0.10	NA	1.00 **±** 0.17	0.99	1.00 **±** 0.08	NA	0.90 **±** 0.09	0.014	1.00 **±** 0.08	NA	1.05 **±** 0.09	0.26
	*penB*	1.00 **±** 0.22	NA	5.04 **±** 1.26	<0.001	1.00 **±** 0.06	NA	1.15 **±** 0.10	<0.001	1.00 **±** 0.10	NA	1.14 **±** 0.10	0.005
	*ampC*	1.00 **±** 0.17	NA	3.41 **±** 0.71	<0.001	1.00 **±** 0.06	NA	0.94 **±** 0.10	0.13	1.00 **±** 0.12	NA	0.93 **±** 0.11	0.16

a Cells of Bu278 (wild type) and its Δ*slt* and Δ*nagZ* mutants were grown to log phase in LB medium. Equal portions of the cell cultures remained untreated or were treated with a subinhibitory concentration of 1 μg/ml IMP, MEM, or CAZ. Total RNA was isolated after an additional 1 h of incubation at 37°C. The penA*, *penB*, *ampC*, and *oxa* mRNA levels were determined by RT-qPCR. Standard deviations (SD) between three biological replicates are indicated. Two-way ANOVA and Sidak’s multiple-comparison test were used to determine the significance of the change in fold mRNA expression levels between treated (MEM, IPM, or CAZ) and untreated strains. *P* values of <0.05 were considered significant. NA, not applicable.

### Only PenB is required for B. ubonensis Bu278 carbapenem resistance.

Because PenB and AmpC expression is highly inducible by carbapenems, we next assessed the potential involvement of these β-lactamases in carbapenem and CAZ resistance using unmarked deletion mutants (Tables 1 to 3). Because the putative class D OXA has never been implicated in clinically significant β-lactam resistance in any *Burkholderia* species, we focused on PenA*, PenB, and AmpC. Bu278 PenA* and AmpC deletion mutants exhibited the same MICs for MEM (≥32 μg/ml), IMP (≥32 μg/ml), CAZ (8 μg/ml), and amoxicillin (AMX; ≥256 μg/ml) as wild-type strain Bu278 (Table 1). In contrast, the PenB deletion mutant became highly susceptible to IMP and MEM, with MICs of 0.19 μg/ml for IMP and 0.75 μg/ml for MEM, which were at least 168- and 43-fold lower than the MIC for Bu278, respectively (MICs, ≥32 μg/ml for both IMP and MEM). The CAZ MIC of the PenB mutant (1.5 μg/ml) was 5-fold lower than that of Bu278 (8 μg/ml). The Δ*penB* mutation was complemented by single-copy expression of wild-type *penB* from its endogenous promoter (Table 2). Consistent with PenB being a class A β-lactamase, the MEM, IMP, and CAZ resistance of Bu278 was completely reversed to a susceptible phenotype by the β-lactamase inhibitor avibactam (AVI). Avibactam has weak intrinsic activity against Bu278, with a measurable MIC of 256 μg/ml (Table 3).

### Burkholderia ubonensis PenB and AmpC exhibit β-lactamase activity in Escherichia coli, but B. ubonensis PenA* does not.

**FIG 2:**
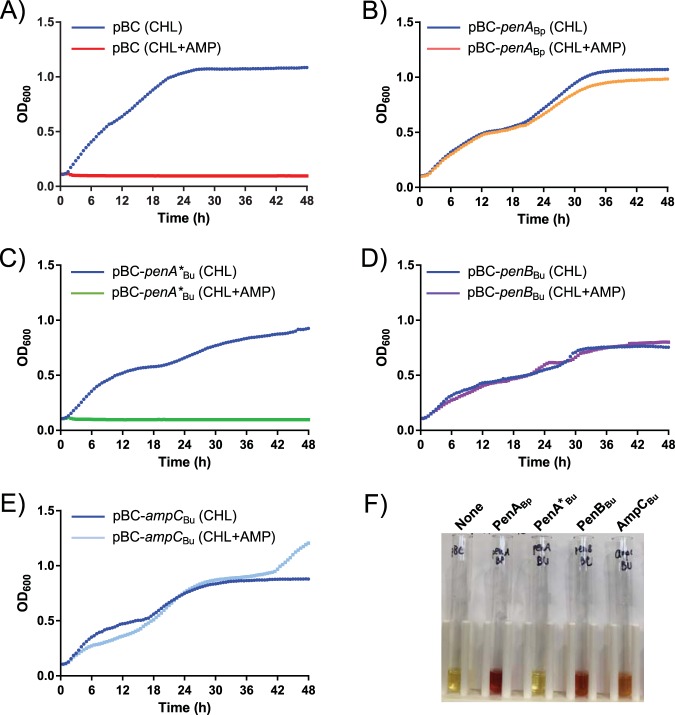
Functional assessment of *Burkholderia* β-lactamases in E. coli. (A to E) Growth curves. Cells of E. coli GBE180 harboring vector pBC-SK(−) (pBC) (A) or recombinant plasmids that constitutively express β-lactamase genes from the *lac* promoter, B. pseudomallei *penA* (*penA*_Bp_) (B) and B. ubonensis Bu278 penA* (penA*_Bu_) (C), *penB* (*penB*_Bu_) (D), and *ampC* (*ampC*_Bu_) (E), were tested. The β-lactamase genes were fused to the E. coli DsbA signal sequence-coding sequence for soluble periplasmic expression. Bacteria were grown at 37°C in LB medium in the presence of chloramphenicol (CHL) for plasmid maintenance or CHL plus 100 μg/ml ampicillin (CHL+AMP). The data shown are the means plus standard deviations of recordings from four adjacent wells. The graphs are representative of those from three repeat experiments. OD_600_, optical density at 600 nm. (F) Qualitative β-lactamase nitrocefin assays. Bacteria containing the same plasmids indicated for panels A to E were grown overnight at 37°C in LB medium-CHL, and periplasmic fluids were obtained via osmotic shock. The picture was taken after nitrocefin hydrolysis at 37°C for 1.5 h by ∼17% of total shock fluid obtained from ∼2.5 × 10^9^ cells of the respective overnight cultures. None, vector only.

Because deletion of neither Bu278 PenA* nor AmpC affected the susceptibility to AMX, we assessed whether these proteins exhibited β-lactamase activity when overexpressed in an Escherichia coli laboratory strain which harbored no endogenous β-lactamases. As positive controls, we included B. pseudomallei PenA (PenA_Bp_) and B. ubonensis PenB (PenB_Bu_). To this end, the native signal sequences of B. ubonensis Bu278 PenA* (PenA*_Bu_), PenB_Bu_, B. ubonensis AmpC (AmpC_Bu_), and PenA_Bp_ were replaced by the E. coli DsbA signal sequence (ssDsbA) for the export of soluble proteins into the periplasm (30). As a readout for β-lactamase expression and activity, we monitored growth in the presence and the absence of ampicillin (AMP) (Fig. 2). E. coli bacteria expressing PenA_Bp_ (Fig. 2B), PenB_Bu_ (Fig. 2D), or AmpC_Bu_ (Fig. 2E) were able to grow in LB-AMP medium. In contrast, E. coli cells containing the empty vector control (Fig. 2A) or expressing PenA*_Bu_ (Fig. 2C) did not grow in the presence of AMP. Periplasmic fractions from strains expressing PenA_Bp_, PenB_Bu_, and AmpC_Bu_, but not fractions from strains from PenA*_Bu_, showed β-lactamase activity in a qualitative nitrocefin assay (Fig. 2F), corroborating the results obtained in the growth experiments whose results are shown in Fig. 2A to E.

### PenA* from Bcc bacteria does not exhibit β-lactamase activity due to conserved active-site mutations.

**FIG 3:**
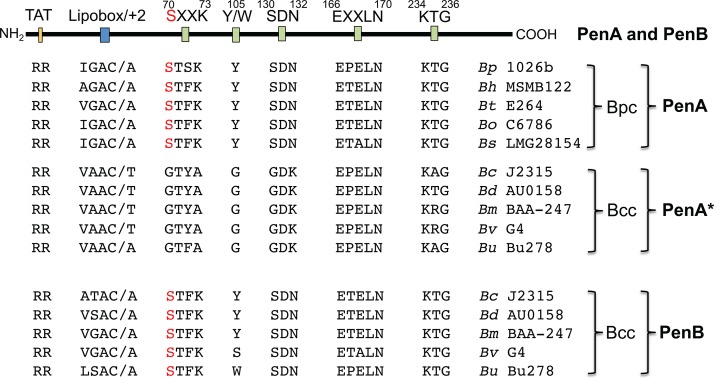
Comparison of class A β-lactamase domain structures in representative Bcc and Bpc bacteria. The schematic domain structure of *Burkholderia* PenA and PenB class A β-lactamases is indicated at the top. It includes the twin arginine transport (TAT) secretion and lipobox/position +2 membrane localization domains, the conserved ^70^SXXK^73^, ^130^SDN^132^, ^166^EXXLN^170^, and ^234^KTG^236^ Ambler motifs, and the tyrosine or tryptophan residue 105 (Y/W^105^). The active-site serine is indicated in red font. Most B. pseudomallei isolates contain a serine at position 72, which confers clavulanic acid sensitivity; a serine 72-to-phenylalanine change results in clavulanic acid resistance. Bpc bacteria contain only PenA, and Bcc bacteria contain both PenA* and PenB. The Bcc bacterial PenA* proteins possess the predicted (proven for B. pseudomallei) TAT secretion and lipobox/position +2 membrane localization domains but lack the ^70^SXXK^73^ and ^130^SDN^132^ motifs and the Y/W^105^ residue. Abbreviations: *Bc*, B. cenocepacia; *Bd*, B. dolosa; *Bh*, B. humptydoonesis; *Bm*, B. multivorans; *Bo*, B. oklahomensis; *Bp*, B. pseudomallei; *Bs*, B. singularis; *Bt*, B. thailandensis; *Bu*, B. ubonensis; *Bv*, B. vietnamiensis. The coordinates for the B. dolosa AU0158 KTG motif are residues 235 to 237.

It was recently noted that B. multivorans PenA* lacks two Ambler consensus sequences, including the active-site serine (23). To assess whether this is a conserved trait in Bcc bacteria, we examined the PenA* sequences from representative bacteria of five Bcc species: B. cenocepacia, B. dolosa, B. multivorans, B. vietnamiensis, and B. ubonensis (Fig. 3). Of the four Ambler motifs that contain critical active-site residues and the conserved tyrosine or tryptophan at position 105 (Y/W^105^), only the ^166^EXXLN^170^ motif and a noncanonical ^234^KTG^236^ motif [^234^K(T/R/A)G^236^] were present in PenA* from these bacteria. The ^70^SXXK^73^ motif, where S^70^ is the active-site serine, and the ^130^SDN^132^ motif, as well as the Y/W^105^ motif, were missing. While these motifs/residues are lacking, their respective replacements were remarkably conserved. In contrast to PenA*, the four Ambler motifs and, with exception of B. vietnamiensis G4, Y/W^105^ were conserved in the respective PenB homologs. The export signal (the twin arginine residues of the twin arginine transport [TAT] system) and the membrane localization signals (the lipobox and the putative OM sorting signal at the +2 position) were conserved among all PenA, PenA*, and PenB proteins. PenA TAT secretion (17) and membrane localization (31) have previously been shown for B. pseudomallei PenA. The presence of amino acids other than aspartic acid in the +2 position of the mature proteins (with a modified cysteine at the +1 position) suggests that all are OM-localized lipoproteins. This paradigm is based on E. coli data (32, 33), but a recent study with P. aeruginosa suggests that this may not always be the case with other bacteria (34).

**FIG 4:**
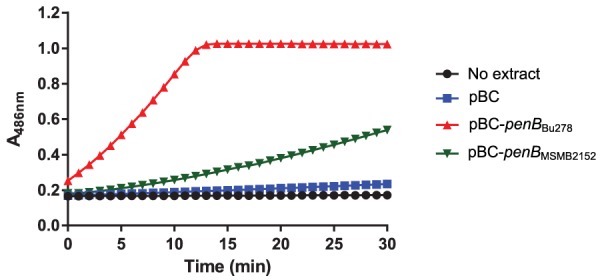
PenB proteins from meropenem-resistant and -susceptible B. ubonensis strains exhibit differential activity. Cell extracts (CFE) were prepared from E. coli GBE180 cells harboring vector pBC-SK(−) (pBC) or recombinant plasmids that constitutively express the *penB*_Bu278_ and *penB*_MSMB2521_ β-lactamase genes from the *lac* promoter. Nitrocefin hydrolysis in reaction mixtures with no extract addition or addition of 5 μg of CFE protein from strains expressing no β-lactamase (pBC), PenB_Bu278_ (pBC-*penB*_Bu278_), or PenB_MSMB2152_ (pBC-*penB*_MSMB2152_) was recorded for 30 min at 37°C. The results shown are representative of those from three experiments conducted on separate days.

These observations are consistent with the findings that PenB is a β-lactamase (Fig. 2 and 4) and that B. ubonensis PenA* lacks β-lactamase activity (Fig. 2). Due to the conserved nature of the PenA* proteins, this is likely also true for other Bcc species. These observations also suggest that the PenA* protein is exported via the TAT system and localized to the OM.

### PenB from a highly carbapenem-resistant B. ubonensis strain confers increased resistance to a susceptible B. ubonensis strain.

Although MEM^r^ in B. ubonensis is not uncommon, strains from diverse geographical sources, including the Northern Territory of Australia and Puerto Rico, exhibit a wide range of susceptibilities (MICs, 2 μg/ml to ≥32 μg/ml) (21, 35). To assess whether PenB from the highly carbapenem-resistant strain Bu278 was sufficient to bestow resistance on the carbapenem-susceptible B. ubonensis strain MSMB2152, we examined the relative contributions of PenB from strains Bu278 (PenB_Bu278_) and MSMB2152 (PenB_MSMB2152_) to MEM and IMP resistance in the Bu278 and MSMB2152 Δ*penB* derivatives Bu312 and Bu410, respectively (Table 6). When expressed from the native *penB* promoter in a single copy in the Δ*penB*_Bu278_ strain Bu312, PenB_Bu278_ (complemented strain Bu399) and PenB_MSMB2152_ (complemented strain Bu393) restored MEM and IMP resistance to levels equivalent to those observed in the respective Bu278 (MICs, ≥32 μg/ml for MEM and IMP) and MSMB2152 (MICs, 3 μg/ml for MEM and 6 μg/ml for IMP) parental strains. Expression of PenB_MSMB2152_ in the Δ*penB*_MSMB2152_ strain Bu410 resulted in levels of MEM^r^ and IMP resistance (IMP^r^) equivalent to those observed in MSMB2152; i.e., MICs of 3 μg/ml (MEM) and 8 μg/ml (IMP) in the complemented mutant (Bu412) versus MICs of 3 μg/ml (MEM) and 12 μg/ml (IMP) in MSMB2152. Expression of PenB_Bu278_ in Bu410 increased the MEM^r^ level 4-fold over that observed in MSMB2152 (MICs for Bu414 and MSMB2152, 12 μg/ml and 3 μg/ml, respectively); IMP^r^ was increased 2.7-fold (MICs for Bu414 and MSMB2152, 32 μg/ml and 12 μg/ml, respectively).

**TABLE 6:** Role of PenB in carbapenem susceptibility of B. ubonensis strains

Strain	Relevant genotype	PenB expressed	MIC (μg/ml)^*a*^	
			MEM	IMP
Bu278	Wild type	PenB_Bu278_	≥32	≥32
MSMB2152	Wild type	PenB_MSMB2152_	3	12
Bu312	Bu278 Δ*penB*		0.75	0.19
Bu410	MSMB2152 Δ*penB*		0.5	0.25
Bu397	Bu278 Δ*penB glmS3*::mini- Tn*7*T		0.75	0.19
Bu399	Bu278 Δ*penB glmS3*::mini- Tn*7*T-*P_penB_-penB*_Bu278_*^+^*	PenB_Bu278_	32	≥32
Bu393	Bu278 Δ*penB glmS3*::mini- Tn*7*T-*P_penB_-penB*_MSMB2152_*^+^*	PenB_MSMB2152_	3	6
Bu416	MSMB2152 Δ*penB* *glmS1*::mini-Tn*7*T		0.5	0.25
Bu414	MSMB2152 Δ*penB glmS1* mini-Tn*7*T-*P_penB_-penB*_Bu278_*^+^*	PenB_Bu278_	12	32
Bu412	MSMB2152 Δ*penB* *glmS1*::mini-Tn*7*T-*P_penB_*- *penB*_MSMB2152_*^+^*	PenB_MSMB2152_	3	8

a The MIC was determined using the Etest method, performed in triplicate on three separate days, and values are reported as the mode of the readings.

The different carbapenem resistance levels observed in strains Bu278 and MSMB2152 were not attributable to the increased transcription of *penB* because *penB* induction levels in strain Bu278 were 2-fold lower after MEM challenge and the same after IMP challenge (Table S3). When expressed in E. coli, PenB_Bu278_ hydrolyzed nitrocefin more rapidly than PenB_MSMB2152_ (Fig. 4). However, absent a full kinetic evaluation of the two enzymes, these data are simply indicative of the possibility that PenB_Bu278_ is perhaps a more robust β-lactamase than PenB_MSMB2152_ and that this property may be a factor contributing to the significantly higher MEM and IMP resistance observed in Bu278 than in MSMB2152. Even so, the incomplete restoration of MEM^r^ in the Δ*penB*_MSMB2152_ strain upon PenB_Bu278_ expression (strain Bu414; MIC, 12 μg/ml) to the level observed in Bu278 (MIC, ≥32 μg/ml) hints at the contribution of other factors to the high-level MEM^r^ observed in Bu278 (Table 6). Curiously, while IMP and MEM *penB* induction levels in Bu278 and MSMB2152 were either the same (IMP) or reduced 2-fold (MEM), *ampC* induction levels after IMP and MEM challenge were significantly higher in MSMB2152 than in Bu278 (7- and 11-fold, respectively) (Table S3). The significance of these higher AmpC expression levels as a consequence of IMP and MEM challenge is unclear.

10.1128/mBio.00592-20.6 TABLE S3 B. ubonensis meropenem-resistant and -susceptible strains express high *penB* transcript levels. Download Table S3, PDF file, 0.1 MB. Copyright © 2020 Somprasong et al. 2020 Somprasong et al. This content is distributed under the terms of the Creative Commons Attribution 4.0 International license.

### PenR-dependent PenB_Bu_ induction in Burkholderia thailandensis in response to β-lactam challenge.

**FIG 5:**
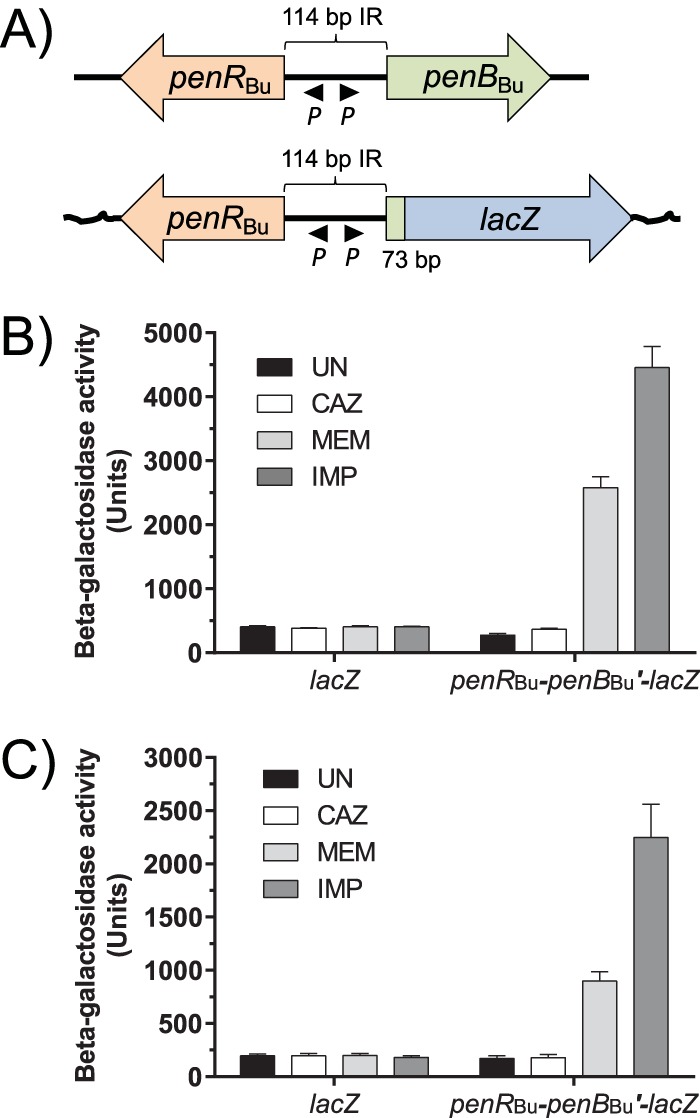
B. ubonensis PenB β-lactamase expression is inducible in B. thailandensis. (A) Organization of the *penR-penB* region of B. ubonensis. Transcription of the Bu278 *penB*_Bu_ gene is under transcriptional control of the LysR-type transcriptional regulator PenR_Bu_, encoded by Bu278 *penR* (*penR*_Bu_). (Top) The *penR*_Bu_ and *penB*_Bu_ genes are transcribed from predicted adjacent divergent promoters (*P*) located within the 114-bp *penR*_Bu_*-penB*_Bu_ intergenic region (IR). (Bottom) A mini-Tn*7* element that contains *penR*_Bu_-IR-*penB*_Bu_*′-lacZ*, where *penB*_Bu_*′-lacZ* is a transcriptional fusion containing the first 73 bp of *penB*_Bu_, was constructed. (B and C) β-Galactosidase (β-Gal) activities of the *penB*_Bu_*′-lacZ* fusions. Mini-Tn*7* elements containing the empty vector (*lacZ*) or *penR*_Bu_-IR-*penB*_Bu_*′-lacZ* were integrated into B. ubonensis strain Bu333, a gentamicin-susceptible Bu278 derivative (B), or the B. pseudomallei surrogate B. thailandensis strain Bt36 (C), and β-Gal expression was measured in uninduced cells (UN) or cells induced for 1 h with subinhibitory concentrations (1 μg/ml) of CAZ, IMP, or MEM. β-Gal activities were determined and are expressed in Miller units. Data are shown as the means from two biological replicates in technical triplicate, with error bars representing 1 standard deviation.

As mentioned above, the PenA β-lactamase is not inducible in Bpc bacteria upon β-lactam challenge, and these bacteria do not possess the equivalents of PenR and PenB present in Bcc bacteria. To assess whether Bpc bacteria would be able to express the PenB β-lactamase in response to β-lactam challenge, especially MEM and IMP, as a result of peptidoglycan synthesis perturbation, we chose the closely related and widely used B. pseudomallei surrogate B. thailandensis. To avoid potential complications by the endogenous PenA, we elected to not use PenB β-lactamase activity as a readout but, rather, used *penB′-lacZ* transcriptional activity. To this end, we constructed a mini-Tn*7* element for chromosomal integration of a *penB*_Bu278_*′-lacZ* transcriptional fusion under the control of PenR of strain Bu278 (Fig. 5A) and assessed β-galactosidase (β-Gal) expression in B. ubonensis (Fig. 5B) and B. thailandensis (Fig. 5C). β-Gal expression showed the same patterns in B. ubonensis and B. thailandensis; i.e., it was inducible by MEM and IMP but not by CAZ, although the latter demonstrated significant inducibility of *penB*_Bu_ when mRNA levels in cells challenged with the same CAZ concentration were quantitated (Table 4).

## DISCUSSION

Infections caused by B. pseudomallei, one of two known pathogenic members of the Bpc, are increasingly reported in parts of the world where the bacterium and the disease that it causes, melioidosis, have traditionally been underreported (1, 2). Acquired antimicrobial resistance is rare in B. pseudomallei (7, 36) and, in the absence of any demonstrated horizontal resistance gene transfer, is confined to genomically encoded determinants whose expression or activity is altered by mutational events (8–16, 37, 38). However, due to the dearth of clinically useful antibiotics, any resistance affecting their use has severe and potentially fatal consequences. Because of the natural colistin resistance of *Burkholderia* species (2, 24), MEM is the drug of choice and last resort for patients that are afflicted with severe cases of melioidosis (2, 6). High-level MEM^r^ has not yet been reported in B. pseudomallei, but decreased susceptibility to MEM occurring over the course of melioidosis therapy has been documented (16). However, the recent discovery that highly MEM^r^ strains of B. ubonensis, a nonpathogenic Bcc member, are frequently coisolated from the environment with B. pseudomallei raised the specter of potential horizontal resistance gene transfer between the two species (21).

Our studies of B. ubonensis MΕM^r^ mechanisms presented here revealed features that are shared with other Bcc bacteria, but they also revealed novel aspects of β-lactam resistance in *Burkholderia* species and even aspects that are specific to B. ubonensis. In addition, while our collective knowledge of β-lactam resistance in Bcc bacteria has, for the most part, been derived from studies conducted with different species, our study focused on B. ubonensis. Not surprisingly, the mode of regulation of the cadre of β-lactamases encoded by B. ubonensis is similar to that of the β-lactamases encoded by other Bcc bacteria. This bacterium encodes three potential β-lactamases, one class A enzyme (PenB), one class C enzyme (AmpC), and one OXA-like class D enzyme. Although B. ubonensis genome annotations propose a second class A β-lactamase, PenA, a homolog of B. pseudomallei PenA, we show in this study that this protein is not a β-lactamase and we thus call it PenA*. By comparison, B. pseudomallei encodes one class A β-lactamase (PenA) and the OXA-like class D β-lactamase, but PenA is the only clinically significant enzyme. Like the respective enzymes in B. cenocepacia (28) and B. multivorans (23), the expression of PenB_Bu_ and AmpC_Bu_ is coregulated and induced at the transcriptional level in a PenR-dependent manner in response to challenge with subinhibitory concentrations of β-lactams, which, in our studies, were limited to the clinically most relevant IMP, MEM, and CAZ. Like B. pseudomallei PenA (17), B. ubonensis PenA* is not inducible by these antibiotics, and neither is OXA. We demonstrated that the periplasmic soluble lytic transglycosylase (Slt) and the cytoplasmic β-*N*-acetylhexosaminidase (NagZ) play crucial roles in the β-lactamase induction process in response to β-lactam challenge. PenB_Bu_ and AmpC_Bu_ expression was not completely dependent on Slt and NagZ, suggesting that, like in the *Enterobacteriaceae* and P. aeruginosa, other factors play a significant role in the induction of PenB_Bu_ and AmpC_Bu_ in response to β-lactam challenge (26, 27, 39).

Mutational analyses confirmed that PenB_Bu_ is a carbapenemase and the main factor for high-level carbapenem resistance in strain Bu278 and that this resistance can be completely reversed by the β-lactamase inhibitor avibactam, which by itself lacks significant antimicrobial activity against B. ubonensis. The same studies also revealed that in B. ubonensis, PenB has weak activity against CAZ, which is not sufficient for clinically significant CAZ resistance (assuming that the breakpoints for CAZ susceptibility in B. ubonensis and B. pseudomallei are similar, i.e., ≤8 μg/ml). Finally, deletion of PenB slightly affected the AMX MIC. Although PenA* and AmpC seemed to have no significant activity against carbapenems and CAZ and their deletion did not affect AMX MICs, the data are more difficult to interpret because the MIC data on which these observations are based have no endpoints. However, when expressed in the E. coli periplasm, all enzymes except PenA* showed β-lactamase activity in a nitrocefin assay and supported growth in AMP-containing medium. These and previously published data for B. multivorans suggest that, upon induction, AmpC could have a minor effect on MICs (23). A notable difference between the two studies is that in our study the expression of B. ubonensis AmpC in E. coli supported bacterial growth in the presence of 100 μg/ml AMP, whereas the expression of B. multivorans AmpC conferred significantly lower resistance (MIC, 8 μg/ml) on E. coli (23).

Examination of the amino acid sequences of the PenA, PenA*, and PenB enzymes of representative species of the Bcc and Bpc revealed some novel findings about the properties of these proteins. Bpc PenA and Bcc PenB contain the Ambler motifs and the Y/W^105^ residue and are functional β-lactamases. In contrast, and as previously noted but not experimentally shown for B. multivorans (23), the Bcc PenA* proteins lack the ^70^SXXK^73^ and ^130^SDN^132^ motifs, as well as residue Y^105^, and contain a noncanonical ^234^K(T/R/A)G^236^ motif. A lack of critical motifs, including the active-site serine 70, explains the lack of β-lactamase activity. A unique feature present only in B. ubonensis and not in other Bcc or Bpc bacterial class A β-lactamases is the presence of a tryptophan instead of a tyrosine at position 105 in all examined strains, irrespective of their MEM^r^ levels. The role of residue 105 in β-lactam binding was previously recognized. For instance, the ability of class A enzymes from other *Burkholderia* species containing tyrosine at position 105 to hydrolyze carbapenems is dependent on the tyrosine conformation in the protein (40). Furthermore, previous studies showed the importance of W105 in the catalytic activity of Klebsiella pneumoniae KPC-2 carbapenemase; its presence overcomes the possible constraints posed by tyrosine at this position (41). In this context, it is noteworthy that B. ubonensis seems to be the only Bpc or Bcc species whose class A β-lactamase active-site residues, i.e., S^70^, W^105^, N^132^, E^166^, R^220^, and T^237^, are identical to those of KPC-2 (42).

We do not yet know why Bcc bacteria maintain PenA*, but several key observations provide the basis for further investigations. First, even though the PenA* derivatives lack two of four Ambler motifs and the Y^105^ that is present in PenA proteins from Bpc bacteria, the substitute motifs are very conserved. This likely serves to maintain the overall structure of the protein, including what constitutes the former active-site pocket (see Fig. S2 in the supplemental material). Second, we previously established that in Bpc bacteria *penA* is the second gene in an operon with *nlpD1* (15), and this arrangement is conserved in Bcc bacteria (Fig. S1). NlpD1 is one of two potential periplasmic cell wall hydrolytic amidase activator proteins with properties similar to those of E. coli NlpD, including being an OM lipoprotein (43). The NlpD1 domain organization in B. pseudomallei and B. ubonensis is conserved (data not shown), and therefore, the NlpD1 proteins from both species likely exhibit a similar function(s). As previously stated, we do not yet understand whether the transcriptional association of *penA* with *nlpD1* is coincidental or implies a functional relationship of the two gene products (15). We hypothesize that the conserved differences in PenA* from Bcc and PenA from Bpc bacteria can be exploited for the development of diagnostic tools for the rapid distinction between the two bacterial complexes.

10.1128/mBio.00592-20.3 FIG S2 Structures of B. pseudomallei PenA and B. vietnamiensis PenA*. Download FIG S2, PDF file, 2.5 MB. Copyright © 2020 Somprasong et al. 2020 Somprasong et al. This content is distributed under the terms of the Creative Commons Attribution 4.0 International license.

Because the PenB enzymes from all examined B. ubonensis strains possess identical active-site residues irrespective of their MEM^r^ levels, the question arises whether PenB is the main carbapenem resistance determinant or whether there are additional factors that determine high versus low MEM^r^ levels in diverse B. ubonensis strains. Comparisons of *penB* expression in a highly MEM^r^ strain (Bu278) and an MEM-susceptible (MEM^s^) strain (MSMB2152) by reverse transcription-quantitative PCR (RT-qPCR) showed that the differential susceptibility was not attributable to the differential expression or copy number of PenB in the two strains, as has been observed in B. pseudomallei (13, 15, 38). Rather, expression of PenB from the highly carbapenem-resistant strain Bu278 in the carbapenem-susceptible strain MSMB2521 and vice versa indicated that intrinsic properties of PenB_Bu278_ contribute significantly to the carbapenem resistance of Bu278. This is rather surprising, given that both PenB_Bu278_ and PenB_MSMB2152_ contain 297 amino acids, of which 97% are identical, including all Ambler motifs and active-site residues. The two proteins differ by 9 amino acids that are distributed throughout the protein. None of the amino acid changes affect the Ω loop, which is notorious for acquiring single amino acid substitutions that expand the substrate spectrum of class A β-lactamases (44), and none of them are closely associated with any Ambler sequence. The more robust β-lactamase activity of PenB_Bu278_ and PenB_MSMB2152_ observed in E. coli is consistent with the notion that the PenB amino acid sequence variation observed between the two strains contributes to differential enzyme activity. Although PenB is a significant contributor to the high carbapenem resistance of strain Bu278, our data suggest that there are additional factors that determine high versus low resistance levels in diverse B. ubonensis strains.

In P. aeruginosa, the OM protein OprD is a major factor of carbapenem resistance (45). Outer membrane proteins are likely to play a role in B. ubonensis MEM^r^, since the random transposon mutagenesis approach identified two independent insertions in the gene encoding the OM protein assembly factor BamC (46), which led to a substantially reduced MEM^r^ (MIC range, ≥32 μg/ml in the parent to 8 μg/ml in the *bamC* mutant) (Table S2). We have identified a putative B. ubonensis OprD homolog (CJO66_RS28785 in Bu278) with 36% identity to P. aeruginosa OprD, but we have not yet found any compelling differences in the OprD amino acid sequences from MEM^r^ and MEM^s^ strains. Other factors that alter OM properties are also known to affect antimicrobial susceptibility. For instance, in *Burkholderia* species, hopanoids were shown to play important roles in resistance to antimicrobials and diverse environmental stresses by strengthening the OM (47, 48). It was recently shown that the HpnN hopanoid efflux transporter plays a role in the intrinsic antimicrobial resistance of B. thailandensis and B. multivorans, presumably by shuttling hopanoids from the cytoplasmic membrane to the OM (49). Hopanoids seem to be a factor contributing to B. ubonensis Bu278 MEM^r^. Transposon mutagenesis identified a Bu278 mutant with an insertion in a VacJ family lipoprotein (CJO66_RS29205), which reproducibly lowered the MIC from ≥32 μg/ml in the parent to 24 μg/ml in the mutant (Table S2). We now know this lipoprotein as HpnM; it is encoded by the second gene in the *hpnN-hpnM* operon, is likely localized to the OM, and is required for efflux pump function (E. W. Yu and H. P. Schweizer labs, unpublished data). As previously shown for B. pseudomallei (15), our data demonstrate that, in addition to PenB expression in response to β-lactam-mediated peptidoglycan synthesis perturbation, other mechanisms contribute to intrinsic B. ubonensis β-lactam resistance.

Lastly, by demonstrating the inducible expression of a PenR-regulated *penB*_Bu_′-*lacZ* fusion in B. thailandensis in response to β-lactam challenge, we showed that although Bpc bacteria seemingly do not encode an inducible β-lactamase, they do possess the key players of the bacterial response to aberrant peptidoglycan synthesis resulting from β-lactam challenge. These must include enzymes and transport mechanisms that provide the intracellular ligands required for PenR-dependent activation of *penB* promoter expression. The inducibility of PenB in B. thailandensis indicates that enhancement of the resistance repertoire of pathogenic Bpc bacteria by the natural acquisition of DNA from drug-resistant near-neighbor species may indeed be of concern, and this topic warrants further study and monitoring. In this context, it is important to note that because PenB is a class A β-lactamase, its activity can be completely suppressed by avibactam.

**FIG 6:**
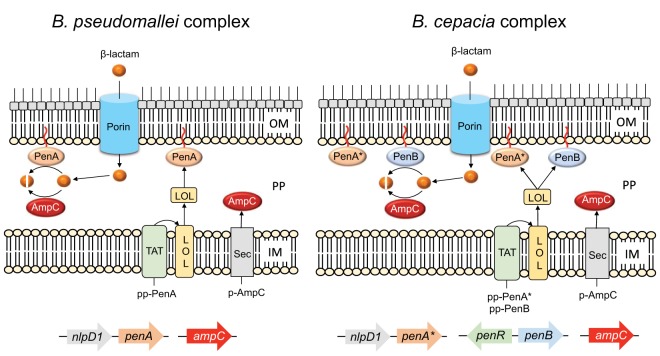
Summary of β-lactamases and their predicted genetic and molecular properties in Bpc and Bcc bacteria. *Burkholderia* species produce at least three active β-lactamases, two class A enzymes (PenA and PenB), and one class C enzyme (AmpC). PenA and PenB are lipoproteins that are (i) synthesized as pre-pro (pp)-proteins, (ii) exported across the inner membrane (IM) to the periplasm (PP) by the twin arginine transport (TAT) system, (iii) lipidated and processed by signal peptidase II, and (iv) likely sorted to the OM by the localization of lipoproteins (LOL) system. AmpC is synthesized as a pre (p)-protein, which, after export via the general secretion pathway (Sec), is processed by signal peptidase I and which then resides in the periplasm as a soluble protein. Bpc bacteria possess only PenA. Bcc bacteria possess both PenA* and PenB. Although all Bcc PenA* proteins analyzed possess the TAT secretion and lipidation export and membrane localization signals, the proteins are not functional β-lactamases because they lack two of the four Ambler motifs, including the active-site serine. In Bcc bacteria, the active class A serine β-lactamase is PenB. Some β-lactams are also cleaved by AmpC, which is present in all Bcc bacteria. It is present in a few Bpc bacteria but is absent from its major representatives, including B. pseudomallei, B. mallei, and B. thailandensis. Unlike PenB and AmpC, which are induced in Bcc bacteria after challenge with some β-lactams, PenA* and PenA expression is not inducible in Bcc and Bpc bacteria, respectively. In B. pseudomallei, it has been shown that *penA* is transcribed at low levels from the promoter of the upstream *nlpD1* gene and that the ensuing transcript levels are sufficient for resistance to penicillin antibiotics. Extended-spectrum β-lactam resistance, e.g., CAZ^r^, requires PenA overexpression or amino acid changes as the result of acquired regulatory and structural mutations. The PenA secretion pathway and membrane (likely OM) localization have been experimentally confirmed in B. pseudomallei and predicted with the SignalP (version 5.0) server (http://www.cbs.dtu.dk/services/SignalP/) and the TatP (version 1.0) server (http://www.cbs.dtu.dk/services/TatP/) for all proteins in Bcc bacteria.

In conclusion, we showed that the repertoire of β-lactamases in B. ubonensis is the same as that in other Bcc bacteria and confirmed that β-lactam resistance in Bcc bacteria like B. ubonensis is distinct from that in Bpc bacteria like B. pseudomallei (40). A fundamental difference between PenB-mediated β-lactam resistance in Bcc bacteria and PenA-mediated β-lactam resistance in Bpc bacteria is the mode by which their expression is governed. PenB expression in Bcc bacteria is inducible in response to β-lactam challenge. In contrast, mutational events affecting PenA expression and the amino acid sequence govern PenA expression in Bpc bacteria like B. pseudomallei, which explains the rarity of β-lactam resistance observed in clinical and environmental B. pseudomallei isolates. In contrast, there are many similarities between Bpc PenA and Bcc PenB. Both are class A β-lactamases that are exported via the TAT system and localized to the membrane after lipidation; the latter two events rarely occur in combination in Gram-negative bacteria. Lastly, it must be emphasized that although it is annotated in sequenced Bcc bacterial genomes as a β-lactamase, PenA* of Bcc bacteria lacks the β-lactamase hydrolytic function and must therefore be distinguished from this family of enzymes. Figure 6 summarizes the β-lactamases and their predicted genetic and molecular properties in Bpc and Bcc bacteria.

## MATERIALS AND METHODS

### Strains, media, and growth conditions.

Burkholderia ubonensis strain Bu278, also known as Bp8955, is a soil isolate from Juncos, Puerto Rico, that is MEM^r^ (MIC, ≥32 μg/ml) (21). Strain MSMB2152 is a soil isolate from the Northern Territory, Australia, and MEM susceptible (MIC, 2 to 3 μg/ml) (21). Mutants derived from these strains are listed in Table S1 in the supplemental material. B. thailandensis Bt36 is a Δ(*amrAB-oprA*) derivative of E264 (50). Escherichia coli strains DH5α (51) and its copy number control derivative, GBE180 (52), were used for cloning and expression experiments. E. coli strain RHO3 was employed for interspecies conjugal transfer of plasmids (53). Lennox broth (LB) containing 5-g/liter NaCl was used for the routine growth of bacteria, and cation-adjusted Mueller-Hinton II broth (MHB) or Mueller-Hinton II agar (MHA) medium (Becton, Dickinson and Company, Sparks, MD) was used for antimicrobial susceptibility assays. For PheS-mediated counterselection, B. ubonensis strains were grown on M9 minimal medium (54) agar plates supplemented with 10 mM glucose and 5 mM *p*-chlorophenylalanine (*p*-Cl-Phe; Acros Organics, NJ, USA). Unless otherwise noted, bacteria were grown at 37°C with aeration.

### Antimicrobial susceptibility testing.

Susceptibility assays were performed using the broth microdilution (BMD) method and cation-adjusted Mueller-Hinton II broth (Becton, Dickinson and Company, Sparks, MD, USA), following Clinical and Laboratory Standards Institute guidelines (55), or Etest, following the manufacturer’s (AB bioMérieux, Marcy l’Etoile, France) guidelines. Ceftazidime (CAZ) was purchased from Sigma-Aldrich, and imipenem (IMP) and meropenem (MEM) were bought from the United States Pharmacopeia (Rockville, MD). Avibactam was purchased from Advanced ChemBlocks (Burlingame, CA, USA).

### Plasmid and mutant construction.

The plasmids used in this study are listed in Table S4. Plasmids were constructed by PCR amplification of target sequences from genomic (Wizard Genomic DNA purification kit; Promega, Madison, WI) or plasmid (NucleoSpin plasmid kit; Macherey-Nagel, Düren, Germany) DNA templates and assembly of the resulting DNA fragments with restriction enzyme-linearized vector sequences using the NEBuilder HiFi DNA assembly master mix (New England Biolabs). Primers were purchased from Integrated DNA Technologies (Coralville, IA), and the sequences are available from the authors upon request.

10.1128/mBio.00592-20.7 TABLE S4 Plasmids used in this study. Download Table S4, PDF file, 0.2 MB. Copyright © 2020 Somprasong et al. 2020 Somprasong et al. This content is distributed under the terms of the Creative Commons Attribution 4.0 International license.

Deletion mutants were constructed using the gene replacement vectors and previously described methods, either pJRC115 and *p*-Cl-Phe counterselection (56) or pEXKm5 or pEDL1005 and either sucrose counterselection (with pEXKm5 and the Δ*slt* mutant only) or I-SceI counterselection (with pEDL1005 and MSMB2152 Δ*penB* only) (53). Plasmid-borne deletions were transferred to B. ubonensis from E. coli mobilizer strain RHO3 as previously described (53). Deletions were verified by PCR and Sanger sequencing of the ensuing DNA fragments. The B. ubonensis deletion mutants are listed in Table S1.

### Deletion mutant complementation.

Deletion strains were complemented using a mini-Tn*7* system, which allows for stable and site-specific single-copy insertions into the bacterial genome (57). The predicted and actual *glmS*-associated *att*Tn*7* sites in B. ubonensis were identified as described in Text S1. Genes originating from strain Bu278 or MSMB2152 were PCR amplified and, using the NEBuilder HiFi DNA assembly master mix. cloned into pTJ1 under *P_BAD_* and AraC or native B. ubonensis *penB* promoter (*P_penB_*) transcriptional control, such that translation was dependent on either an engineered consensus ribosome binding site (RBS) with *P_BAD_* constructs (58) or the resident native RBS with *P_penB_* constructs. The respective mini-Tn*7* expression constructs were transferred to the target B. ubonensis strains via conjugation from E. coli RHO3, along with the empty vector–mini-Tn*7* element, and *glmS*-associated insertions were identified by PCR. The complemented B. ubonensis deletion mutants are listed in Table S1. Although expression from mini-Tn*7*-*P_BAD_* constructs in LB-grown cells was leaky, full induction of gene expression was achieved by addition of 1% l-arabinose (58).

10.1128/mBio.00592-20.1 TEXT S1 Supplemental methods. Download Text S1, PDF file, 0.3 MB. Copyright © 2020 Somprasong et al. 2020 Somprasong et al. This content is distributed under the terms of the Creative Commons Attribution 4.0 International license.

### β-Lactamase expression and secretion in E. coli.

To assess β-lactamase activities in E. coli, the native TAT and lipoprotein signal sequences of B. pseudomallei PenA and B. ubonensis PenA* and PenB, as well as the native signal sequence of B. ubonensis AmpC, were replaced by the E. coli DsbA signal sequence (ssDsbA) (30), as described in Text S1. This placed the hybrid genes under the transcriptional control of *P_lac_* and translational control of a consensus ribosome-binding site, both of which were provided by the vector. Functional expression in E. coli GBE180 was achieved (i) by monitoring the growth at 37°C with shaking in microtiter plates containing LB medium with 25 μg/ml chloramphenicol (CHL) alone for vector maintenance or CHL with 100 μg/ml AMP for cells with plasmids harboring the genes for putative β-lactamases, in which the optical density at 600 nm was read at 30-min intervals in a BioTek Epoch 2 microplate reader (Winooski, VT), and (ii) by assessing comparative β-lactamase expression in cell extracts (CFE). β-Lactamase activity was determined by nitrocefin hydrolysis, recorded as the increase in the absorbance at 486 nm at 37°C on a BioTek Epoch 2 microplate reader. Each 0.2-ml reaction mixture in microtiter plates contained phosphate-buffered saline (pH 7.4), 0.05 mg/ml nitrocefin, and 5 μg of CFE protein. Details are provided in Text S1.

### Transposon mutagenesis.

Transposon mutagenesis of strain Bu278 was performed using Tn*5*-based transposon T23 as previously described (15, 59). Briefly, transformants were selected on LB medium containing 100 μg/ml trimethoprim (TMP). TMP-resistant (TMP^r^) colonies were picked and arrayed into 96-well plates containing LB with 100 μg/ml TMP and 10% glycerol. The plates were incubated for 36 h at 37°C and then stored at −80°C. For determination of MEM susceptibilities, the bacteria were replicated onto freshly prepared LB plates with 8 μg/ml MEM. T23 insertion sites in mutants that did not grow on these plates were identified by self-ligation of NotI-digested genomic DNA fragments, followed by the rescue of plasmids containing the TMP^r^ marker and the *ori* residing on T23 after the transformation of E. coli DH5α and Sanger sequencing of the transposon-genome junctions on rescued plasmids (60). Transposon insertion sites were identified by BLAST searches against the Bu278 shotgun genome sequence (GenBank assembly accession number GCA_002276145.1).

### Construction of a *penB*_Bu_*′-lacZ* fusion and β-galactosidase assays.

For construction of a PenR_Bu_-regulated *penB*_Bu_′-*lacZ* transcriptional fusion, a 1,078-bp DNA fragment containing the 891-bp *penR* gene, the 114-bp *penR-penB* intergenic region (IR), and the first 73 bp of *penB*_Bu_ was amplified from Bu278 genomic DNA. Using the NEBuilder HiFi DNA assembly master mix, this PCR fragment was then cloned into pUC18T-mini-Tn*7*T-*lacZ*-Gm to create pPS3458 containing *penR*-IR-*penB′-lacZ* on a mini-Tn*7* element. This mini-Tn*7* element was then transposed into the B. ubonensis strain Bu333 (a gentamicin-susceptible Bu278 *amrB*::T23 mutant; Text S1) or the B. thailandensis Bt36 genome along with the empty vector control. Transformants that contained mini-Tn*7*-*lacZ* or mini-Tn*7*-*penR*-IR-*penB′-lacZ* integrated at the respective *glmS*-associated *att*Tn*7* sites (*glmS3* for Bu333 and *glmS2* for Bt36) were retained. The β-galactosidase (β-Gal) activity in *Burkholderia* strains harboring chromosomally integrated fusion constructs was measured, and activity units were determined by the Miller method (54), using LB-grown log-phase cultures and SDS-chloroform-permeabilized cells, as previously described (15).

### Reverse transcription-quantitative PCR (RT-qPCR).

The expression levels of the mRNA of the target genes were determined in Bu278, its mutant derivatives, and MSMB2152 grown at 37°C in LB medium to the log phase (optical density at 600 nm = 0.6 to 0.8). Total RNA was isolated using an RNeasy Protect bacteria minikit (Qiagen, Valencia, CA), and cDNA synthesis was performed as previously described, employing the 23S rRNA gene as the housekeeping control. The primer sets used were Bp23S_F and Bp23S_R for the B. pseudomallei 23S rRNA gene (61), P3329 (5′-CATCCTGTATCGGCGTTACG) and P3330 (5′-CATAGCTGCCCGATCGTC) for penA*_Bu_, P3331 (5′-AGTACAGCGACAATGCGG) and P3332 (5′-CGGTATTCAGTTCGGGTTCC) for *penB*_Bu_, P3333 (5′-GATGCGGTATCTCAAGGACTG) and P3334 (5′-GATAGCAGATGACGGGACAAC) for *ampC*_Bu_, and P3335 (5′-TCTCGATCTTCTGGTACTCCC) and P3336 (5′-TTCAGTTCGCCCTCGTTG) for *oxa* of B. ubonensis (*oxa*_Bu_). Expression values were pooled between biological replicates. Using GraphPad Prism software (GraphPad Software, La Jolla, CA), two-way analysis of variance (ANOVA) and Sidak’s multiple-comparison test were used to determine the significance of the difference in fold mRNA expression levels. *P* values of <0.05 were considered significant.
