# Supplementary material for: Burkholderia ubonensis Meropenem Resistance: Insights into Distinct Properties of Class A β-Lactamases in Burkholderia cepacia Complex and Burkholderia pseudomallei Complex Bacteria
Source: mBio. 2020 Apr 14;11(2):e00592-20. doi: 10.1128/mBio.00592-20 (PMC7157819; doi:10.1128/mBio.00592-20)
Supplement: FIG S1 [file mBio.00592-20-sf001.pdf]

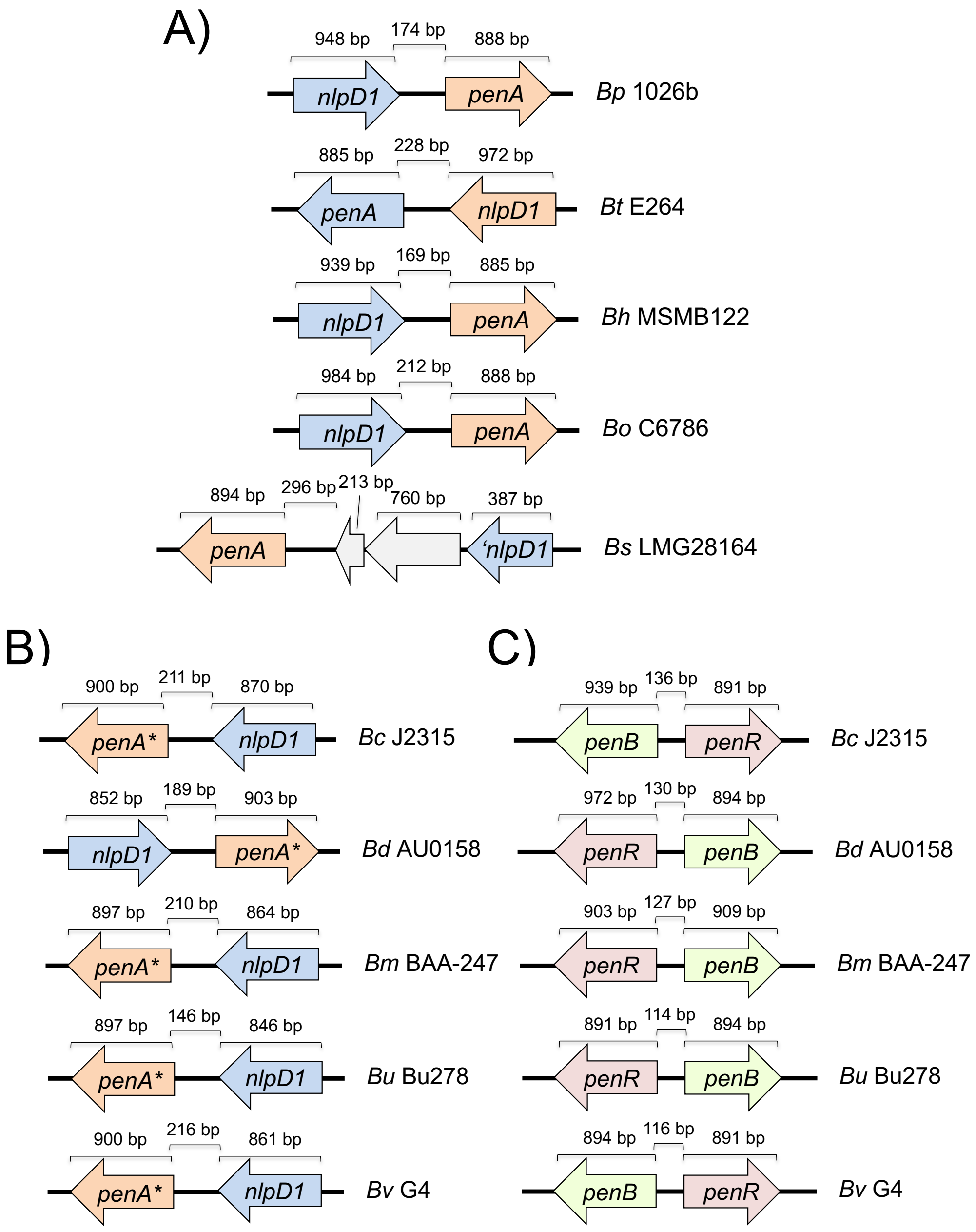

Figure S1. **Genomic organization of the *penA*, *penA** and *penB* loci in *Burkholderia* species. A) The *nlpD1-penA* loci of *B. pseudomallei* complex (Bpc) bacteria** and **B) the *nlpD1-penA** loci of *B. cepacia* complex (Bcc) bacteria.** Based on results obtained with *B. pseudomallei*, the respective *nlpD1* and *penA* or *nlpD1* and *penA** genes are predicted to form an operon (S. Chirakul, M.H. Norris, S. Pagdepanichkit, N. Somprasong, L.B. Randall, J.F. Shirley, B.R. Borlee, O. Lomovskaya, A. Tuanyok, and H.P. Schweizer, Sci Rep 8:10652, 2018, doi:10.1038/s41598-018-28843-7). The lone exception thus far is *B. singularis* (*Bs*) LMG28164 that contains a number of pseudogenes upstream of *penA**, one of which encodes a portion of the 5' *nlpD1* coding sequence. Since only one *B. singularis* genome sequence was available at the time of writing of the current paper it remains unclear whether this scenario is an

anomaly. As discussed in the main manuscript, Bcc bacteria contain *penA** genes that do not express β-lactamase activity. The PenA* proteins possess the twin arginine transport (TAT) secretion and lipobox/+2 localization domains, but lack the SXXK (where the S is the active site serine) and SDN β-lactamase motifs, as well as the conserved ^{105}Y/W residue. NlpD1 is a putative cell wall hydrolytic amidase activator (S. Chirakul, M.H. Norris, S. Pagdepanichkit, N. Somprasong, L.B. Randall, J.F. Shirley, B.R. Borlee, O. Lomovskaya, A. Tuanyok, and H.P. Schweizer, Sci Rep 8:10652, 2018, doi:10.1038/s41598-018-28843-7). **C) The *penR-penB* loci of Bcc bacteria.** The expression of the *penB* β-lactamase gene is under transcriptional control of the LysR-type transcriptional regulator PenR encoded by *penR*. Top: The *penB* and *penR* genes are transcribed from adjacent divergent promoters located within the *penB-penR* intergenic regions. Gene coordinates are according to the *Burkholderia* genome database (www.burkholderia.com; [G.L. Winsor, B. Khaira, T. Van Rossum, R. Lo, M.D. Whiteside, F.S.L Brinkman, Bioinformatics 24:2803-2804, 2008]). Abbreviations: *Bc, B. cenocepacia*; *Bd, B. dolosa*; *Bh, B. humptydooensis*; *Bm, B. multivorans*; *Bo, B. oklahomensis*; *Bp, B. pseudomallei*; *Bs, B. singularis*; *Bt, B. thailandensis*; *Bu, B. ubonensis*; and *Bv, B. vietnamiensis*.
