# Supplementary material for: Burkholderia ubonensis Meropenem Resistance: Insights into Distinct Properties of Class A β-Lactamases in Burkholderia cepacia Complex and Burkholderia pseudomallei Complex Bacteria
Source: mBio. 2020 Apr 14;11(2):e00592-20. doi: 10.1128/mBio.00592-20 (PMC7157819; doi:10.1128/mBio.00592-20)
Supplement: TABLE S1 [file mBio.00592-20-st001.pdf]

Table S1. *Burkholderia ubonensis* strains used in this study

Strain	Description	Source
Bu278[1]	Meropenem resistant soil isolate, Puerto Rico	Price et al., 2017
MSMB2152	Meropenem susceptible soil isolate, Australia	Price et al., 2017
Bu290	Bu278 *slt*::T23	This study
Bu295	Bu278 *nagZ*::T23	This study
Bu296	Bu278 Δ*slt*	This study
Bu308	Bu278 Δ*nagZ*	This study
Bu311	Bu278 Δ*penA*	This study
Bu312	Bu278 Δ*penB*	This study
Bu314	Bu278 Δ*ampC*	This study
Bu333	Bu278 *amrB*::T23	This study
Bu338	Bu278::mini-Tn*7*-TMP^r-*araC*-P_{BAD}[2,3]	This study
Bu352	Bu308::mini-Tn*7*-TMP^r- *araC*-P_{BAD}	This study
Bu356	Bu296::mini-Tn*7*-TMP^r- *araC*-P_{BAD}	This study
Bu373	Bu308::mini-Tn*7*-TMP^r- *araC*-P_{BAD}-*nagZ*$^+$	This study
Bu375	Bu296::mini-Tn*7*-TMP^r- *araC*-P_{BAD}-*slt*$^+$	This study
Bu382	Bu312::mini-Tn7-TMP^r-*araC*[4]	This study
Bu379	Bu312::mini-Tn7-TMP^r-P_{penB}-*penB*$_{Bu278}$$^{+}$[5]	This study
Bu393	Bu312:: mini-Tn*7*T-TMP^r-P_{penB}-*penB*$_{MSMB2152}$$^{+}$	This study
Bu397	Bu312::mini-Tn*7*-TMP^r	This study
Bu399	Bu312::mini-Tn*7*T-TMP^r-P_{penB}-*penB*$_{Bu278}$$^{+}$	This study
Bu410	MSMB2152 Δ*penB*	This study
Bu412	Bu410::mini-Tn*7*T- TMP^r-P_{penB} -*penB*$_{MSMB2152}$$^{+}$[6]	This study
Bu414	Bu410:: mini-Tn*7*T- TMP^r-P_{penB} -*penB*$_{Bu278}$$^{+}$	This study
Bu416	Bu410:: mini-Tn*7*T-TMP^r	This study

[1]Bu278 is also known as Bp8955 (E.P. Price et al., PLoS Negl Trop Dis 11:e0005928, 2017)

[2]Mini-Tn*7* insertions in Bu278 derivatives are located at *glmS3*.

[3]mini-Tn*7* insert from pTJ1 containing a TMP^r marker, the *E. coli araBAD* promoter (P_{BAD}), and *araC* encoding the P_{BAD} activator.

[4]P_{BAD} deleted

[5]$P_{PenB,}$ native *penB* promoter

[6]Mini-Tn*7* insertions in MSMB2152 derivatives are located at *glmS1*.
