# Supplementary material for: Burkholderia ubonensis Meropenem Resistance: Insights into Distinct Properties of Class A β-Lactamases in Burkholderia cepacia Complex and Burkholderia pseudomallei Complex Bacteria
Source: mBio. 2020 Apr 14;11(2):e00592-20. doi: 10.1128/mBio.00592-20 (PMC7157819; doi:10.1128/mBio.00592-20)
Supplement: TABLE S2 [file mBio.00592-20-st002.pdf]

**Table S2.** ***B. ubonensis*** **transposon mutants with increased meropenem susceptibility**

| **Strain** | **MEM MIC (µg/ml)** | **Gene with T23 insertion** |
|---|---|---|
| Bu278 | ≥32 | None (wild-type) |
| Bu284 | 6 | CJO66_RS08510; hypothetical protein |
| Bu285 | 8 | CJO66_RS01305; outer membrane protein assembly factor BamC |
| Bu286 | 6 | CJO66_RS04980; glycosyltransferase family 2 protein |
| Bu288 | 12 | CJ066_RS03065; holo-ACP synthase |
| Bu290 | 1.5 | CJO66_RS20845; lytic soluble transglycosylase, Slt |
| Bu291 | 6 | CJO66_RS25460; endopeptidase La |
| Bu292 | 24 | CJO66_RS29205; VacJ family lipoprotein (HpnM) |
| Bu293 | 24 | CJ066_RS08495; imidazole glycerol phosphate synthase subunit HisF |
| Bu294 | 8 | CJO66_RS01305; outer membrane protein assembly factor BamC |
| Bu295 | 1.5 | CJO66_RS03060; β-*N*-acetylhexosaminidase, NagZ |
