# Supplementary material for: Burkholderia ubonensis Meropenem Resistance: Insights into Distinct Properties of Class A β-Lactamases in Burkholderia cepacia Complex and Burkholderia pseudomallei Complex Bacteria
Source: mBio. 2020 Apr 14;11(2):e00592-20. doi: 10.1128/mBio.00592-20 (PMC7157819; doi:10.1128/mBio.00592-20)
Supplement: TABLE S3 [file mBio.00592-20-st003.pdf]

Table S3. ***B. ubonensis*** **meropenem resistant and susceptible strains express high *penB* transcript levels.**

Strain	Gene	Treatment							
		Untreated		MEM		IMP		CAZ	
		Normalized fold mRNA expression ± SD	*P* value	Normalized fold mRNA expression ± SD	*P* value	Normalized fold mRNA expression ± SD	*P* value	Normalized fold mRNA expression ± SD	*P* value
Bu278	*penA**	1.00 ± 0.08	N/A	0.90 ± 0.16	0.95	0.93 ± 0.22	0.99	1.00 ± 0.19	0.99
	penB	1.00 ± 0.09	N/A	60.61 ± 12.80	<0.001	280.48 ± 53.30	<0.001	4.99 ± 2.08	<0.001
	ampC	1.00 ± 0.08	N/A	14.68 ± 5.73	<0.001	61.46 ± 10.46	<0.001	3.42 ± 1.14	<0.001
MSMB2152	*penA**	1.00 ± 0.16	N/A	0.99 ± 0.15	0.99	0.54 ± 0.14	0.99	0.89 ± 0.23	0.76
	penB	1.00 ± 0.46	N/A	127.99 ± 37.87	<0.001	293.87 ± 79.34	<0.001	3.69 ± 0.90	<0.001
	ampC	1.00 ± 0.19	N/A	160.11 ± 27.66	<0.001	448.35 ± 139.53	<0.001	5.46 ± 1.64	<0.001

Cells of Bu278 (MEM resistant) and MSBM2152 (MEM susceptible) were grown to log phase in LB medium. Equal portions of the cell cultures remained untreated or were treated with sub-inhibitory concentrations of 1 µg/ml IMP, MEM or CAZ. Total RNA was isolated after an additional 1 h incubation at 37°C. The *penA**, *penB* and *ampC* mRNA levels were determined by RT-qPCR. Standard deviations (SD) between three biological replicates are indicated. Two-way ANOVA and Sidak's multiple comparison test were used to determine significance in fold mRNA expression levels between treated (MEM, IPM or CAZ) and untreated strains. *P* values <0.05 were considered significant. N/A, not applicable.
