# Supplementary material for: Burkholderia ubonensis Meropenem Resistance: Insights into Distinct Properties of Class A β-Lactamases in Burkholderia cepacia Complex and Burkholderia pseudomallei Complex Bacteria
Source: mBio. 2020 Apr 14;11(2):e00592-20. doi: 10.1128/mBio.00592-20 (PMC7157819; doi:10.1128/mBio.00592-20)
Supplement: FIG S2 [file mBio.00592-20-sf002.pdf]

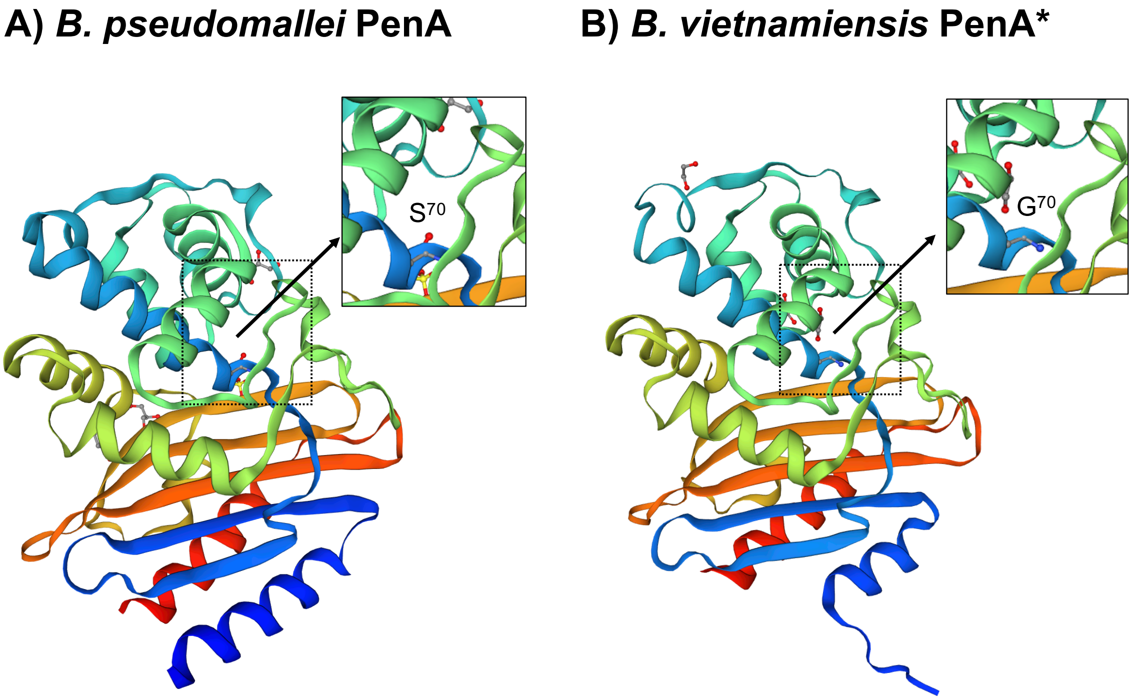

Figure S2. Structures of *B. pseudomallei* PenA (A) and *B. vietnamiensis* PenA* (B). PenA* indicates mutant PenA in which two of four conserved Ambler motifs and Y/W at position 105 are mutated. The boxed area in (**A**) indicates the location of the β-lactam binding pocket of PenA with the active site S^{70} that is changed to G^{70} in inactive PenA* with a similar pocket that lacks many of the critical residues for substrate binding and catalytic activity (**B**). The PenA* amino acid sequence of *B. ubonensis* strain Bu278 was used as query for SWISS-MODEL (https://swissmodel.expasy.org). The primary sequences for *B. pseudomallei* strain K96243 PenA (PDB ID 3W4O; https://www.rcsb.org) (K.M. Papp-Wallace, M.A.Taracila, J.A. Gatta, N. Ohuchi, R.A. Bonomo, and M. Nukaga. 2013. J Biol Chem 288:19090-102, 2013) and *B. vietnamiensis* strain G4 PenA* (PDB ID 5HX9) (Seattle Structural Genomics Center for Infectious Disease [https://www.ssgcid.org]) showed 56.1% and 71.5% identity to *B. ubonensis* strain Bu278 PenA*. The models show that when compared to active PenA β-lactamase the mutational changes resulting in inactive PenA* preserve the overall structure.
