# Supplementary material for: Burkholderia ubonensis Meropenem Resistance: Insights into Distinct Properties of Class A β-Lactamases in Burkholderia cepacia Complex and Burkholderia pseudomallei Complex Bacteria
Source: mBio. 2020 Apr 14;11(2):e00592-20. doi: 10.1128/mBio.00592-20 (PMC7157819; doi:10.1128/mBio.00592-20)
Supplement: TABLE S4 [file mBio.00592-20-st004.pdf]

Table S4. Plasmids used in this study

Name	Description[1]	Source
	**Gene deletion plasmids**	
pEXKm5[2]	KAN^r; allelic exchange vector; sucrose or I-*Sce*I counter-selection	Lopez et al., 2009
pJRC115[3]	TMP^r; allelic exchange vector; *p*-Cl-Phe counter-selection	Chandler et al. 2009
pEDL1005	TMP^r; allelic exchange vector; sucrose or I-*Sce*I counter-selection; constructed by combining a 6,526 bp *Xba*I-*Spe*I fragment from pEXKm5 with a 680 bp *Xba*I fragment with *dhfRII* from pTJ1	This study
pBAD*Sce*-Km	KAN^r; *araC-P_{BAD}-I-sceI* expression vector with pRO1600(Ts) replicon	Lab collection
pPS3435	KAN^r; pEXKm5 with 1,953 bp Δslt_{Bu278} (start to stop codon)	This study
pPS3452	TMP^r; pJRC115 with 897 bp Δ$penA*_{\text{Bu278}}$ (start to stop codon)	This study
pPS3453	TMP^r; pJRC115 with 827 bp Δ$penB_{\text{Bu278}}$ (nt 1-827 of 894 nt ORF)	This study
pPS3454	TMP^r; pJRC115 with 1,176 bp Δ$ampC_{\text{Bu278}}$ (start to stop codon)	This study
pPS3455	TMP^r; pJRC115 with 1,029 bp Δ$nagZ_{\text{Bu278}}$ (start to stop codon)	This study
pPS3531	TMP^r; pEDL1005 with 827 bp Δ$penB_{\text{MSMB2152}}$ (nt 1-827 of 894 nt ORF)	This study
	**Gene fusion plasmids**	
pPS1453[4]	AMP^r, GEN^r; pUC18-mini-Tn*7*T-Gm-*lacZ*, GenBank accession AY599233	Choi & Schweizer
pPS1465[4]	AMP^r; pUC18T-mini-Tn*7*T, GenBank accession AY599230	Choi & Schweizer
pPS3457	AMP^r, GEN^r; pUC18-mini-Tn*7*T-Gm-$penR_{\text{Bu278}}$-IR-$penB_{\text{Bu278}}$'-*lacZ*	This study
pPS3458	AMP^r, GEN^r; pUC18T-mini-Tn*7*T-Gm-$penR_{\text{Bu278}}$-IR-$penB_{\text{Bu278}}$'-*lacZ*	This study
pPS3466	AMP^r, GEN^r; pUC18T-mini-Tn*7*T-Gm-*lacZ*	This study
	**Plasmids for complementation**	
pTJ1[5]	TMP^r; mini-Tn*7* element harboring *araC*-P_{BAD} expression cassette	Damron et al., 2013
pPS3495	TMP^r; pTJ1 without P_{BAD}	This study
pPS3488	TMP^r; pTJ1 with 1,957 bp slt_{Bu278} insert	This study
pPS3487	TMP^r; pTJ1 with 1,031 bp $nagZ_{\text{Bu278}}$ insert	This study
pPS3492	TMP^r; pTJ1 with 1,021 bp $penB_{\text{Bu278}}$ insert[6]	This study
pPS1897	TMP^r, AMP^r; pUC18T-miniTn7T-TMP, GenBank accession DQ493875	Lab collection
pPS3501	TMP^r, AMP^r; pUC18T-miniTn7T-TMP-P_{penB}-$penB_{\text{Bu278}}$	This study
pPS3505	TMP^r, AMP^r; pUC18T-miniTn7T-TMP- P_{penB}-$penB_{\text{MSMB2152}}$	This study
	***E. coli* expression plasmids**	
pBC-SK(-)	CHL^r; *E. coli* cloning and expression vector	Agilent
pGEM-T*Easy*	AMP^r; TA cloning vector	Promega

pPS3482	AMP^r; pGEM-T*Easy* with A-tailed 253 bp gBlocks®[7] fragment containing 47 bp of pBC-SK(-) (including ribosome binding site), 60 bp of 5' *E. coli dsbA* (including $DsbA_{SS}$ and A↓A signal peptidase I cleavage site), and 146 bp of *penA*$_{Bp}$ (codon 9-57 of mature PenA)	This study
pET-24a-'*penA*	KAN^r; pET-24a with 801 bp '*penA*$_{Bp}$ insert from strain 1026b lacking the first 90 nucleotides	This study
pPS3448	CHL^r; pBC-SK(-) with 843 bp *Xba*I-*Bam*HI fragment from pET-24a-'*penA*$_{Bp}$ lacking the first 90 nucleotides	This study
pPS3449	CHL^r; pBC-SK(-) with 809 bp *Nde*I-*Bam*HI fragment containing '*penA*$_{Bu278}$ lacking the first 93 nucleotides	This study
pPS3450	CHL^r; pBC-SK(-) with 800 bp *Nde*I-*Bam*HI fragment containing '*penB*$_{Bu278}$ lacking the first 99 nucleotides	This study
pPS3451	CHL^r; pBC-SK(-) with 1,121 bp *Nde*I-*Bam*HI fragment containing '*ampC*$_{Bu278}$ lacking the first 60 nucleotides	This study
pPS3462	CHL^r; pBC-SK(-) expressing $PenA_{Bp}$ with $DsbA_{ss}$ (*Nde*I+*Bam*HI digested pPS3448 assembled with PCR fragments from pPS3482 and pPS3448)	This study
pPS3468	CHL^r; pBC-SK(-) expressing $PenA*_{Bu278}$ with $DsbA_{ss}$ (*Xba*I+*Bam*HI digested pPS3462 assembled with PCR fragments from pPS3462 and pPS3449)	This study
pPS3469	CHL^r; pBC-SK(-) expressing $PenB_{Bu278}$ with $DsbA_{ss}$ (*Xba*I+*Bam*HI digested pPS3462 assembled with PCR fragments from pPS3462 and pPS3450)	This study
pPS3471	CHL^r; pBC-SK(-) expressing $AmpC_{Bu278}$ with $DsbA_{ss}$ (*Xba*I+*Bam*HI digested pPS3462 assembled with PCR fragments from pPS3462 and pPS3451)	This study
pPS3538	CHL^r; pBC-SK(-) expressing $PenB_{MSMB2152}$ with $DsbA_{ss}$ (*Xba*I+*Bam*HI digested pPS3469 assembled with PCR fragments from pPS3469 and MSMB)	This study

[1]Abbreviations: AMP, ampicillin; *Bp*, *B. pseudomallei*; *Bu*, *B. ubonensis*; CHL, chloramphenicol; GEN, gentamicin; IR, intergenic region; KAN, kanamycin; p-Cl-Phe, p-chloro-phenylalanine; TMP, trimethoprim

[2]C.M. Lopez et al., Appl Env Microbiol 75:6496, 2009

[3]J.R. Chandler et al., J Bacteriol 191:5901, 2009

[4]K.0H. Choi and H.P. Schweizer, Nat Protocols 1: doi:10.1038/nprot.2006.24, 2006

[5]F.H. Damron et al., Appl Env Microbiol 79:718, 2013

[6]P_{BAD} deleted and replaced with the 114 bp *penR*$_{Bu278}$-*penB*$_{Bu278}$ intergenic region containing the *penB*$_{Bu278}$ promoter.

[7]gBlocks® was purchased from Integrated DNA Technologies (Coralville, IA)
