# Supplementary material for: Burkholderia ubonensis Meropenem Resistance: Insights into Distinct Properties of Class A β-Lactamases in Burkholderia cepacia Complex and Burkholderia pseudomallei Complex Bacteria
Source: mBio. 2020 Apr 14;11(2):e00592-20. doi: 10.1128/mBio.00592-20 (PMC7157819; doi:10.1128/mBio.00592-20)
Supplement: TEXT S1 [file mBio.00592-20-s0001.pdf]

Supplemental Methods

1. Establishment of the mini-Tn*7* system in *B. ubonensis* Bu278 (also known as Bp8955)

With few exceptions (K.-H. Choi, and H.P. Schweizer, Nat Protocols 1:170-178, 2006), in Gram-negative bacteria transposon Tn*7* integrates at *att*Tn*7* sites located immediately downstream of the lone *glmS* gene. GlmS is a glutamine-fructose-6-phosphate aminotransferase, an essential enzyme of the essential UDP-*N*-acetyl-glucosamine synthesis pathway (K.-H. Choi, J.B. Gaynor, K.G. White, C. Lopez, C.M. Bosio, R.R. Karkhoff-Schweizer, and H.P. Schweizer, Nat Methods 2:443-448, 2005). In contrast, most *Burkholderia* species contain multiple *glmS* genes, only one of which is essential, and thus multiple *att*Tn*7* sites. The number ranges from one in *B. gladioli* pathovar *cocovenenans* (N. Somprasong, I. McMillan, R.R. Karkhoff-Schweizer, S. Mongkolsuk, and H.P. Schweizer, BMC Res Notes 3:308, 2010), two in *B. mallei* (K.-H. Choi, D. DeShazer, and H.P. Schweizer, Nat Protocols 1:162-169, 2006) and *B. thailandensis* (K.-H.Choi, J.B. Gaynor, K.G. White, C. Lopez, C.M. Bosio, R.R. Karkhoff-Schweizer, and H.P. Schweizer, Nat Methods 2:443-448, 2005) and three in *B. pseudomallei* (K.-H. Choi, T. Mima, Y. Casart, D. Rholl, A.Kumar, I.R. Beacham, and H.P. Schweizer, Appl Env Microbiol 74:1064-1075, 2008).

A search of the *B. ubonensis* Bp8955 draft genome (NCBI Bioproject PRJNA340345) and the *Burkholderi*a Genome Database (www.burkholderia.com) (G.L. Winsor, B. Khaira , T. Van Rossum , R. Lo , M.D. Whiteside, and F.S.L. Brinkman, Bioinformatics 24:2803-2804, 2008) identified four *glmS* genes that we named *glmS1* to *glmS4* (**Fig. 1**). To ascertain integration of Tn*7* into any of the putative *glmS*-associated *att*Tn*7* sites 150 ng of a mini-Tn*7* delivery vector containing a mini-Tn*7* element with a trimethoprim (TMP) resistance marker and 150 ng of pTNS3 containing the genes for the site-specific TnsABCD transposition pathway were co-electroporated into *B. ubonensis* strain Bu278 as previously described (K.-H. Choi, T. Mima, Y. Casart, D. Rholl, A.Kumar, I.R. Beacham, and H.P. Schweizer, Appl Env Microbiol 74:1064-1075, 2008). Transformants were selected on LB medium supplemented with 100 µg/ml TMP. Transposon insertions sites were mapped using a primer annealing to a Tn*7* left end sequence (P_{Tn7L}) and species-specific primers annealing to sequences downstream of the respective *glmS* genes ($P_{BUGlmS1}$ - $P_{BUGLMS4}$) (**Table**) using previously described strategies and methods (K.-H. Choi, J.B. Gaynor, K.G. White, C. Lopez, C.M. Bosio, R.R. Karkhoff-Schweizer, and H.P. Schweizer, Nat Methods 2:443-448, 2005;K.-H. Choi, and H.P. Schweizer, Nat Protocols 1:153-161, 2006). (Note: To avoid issues with sequence variations occurring downstream of *glmS* in other *B. ubonensis* strains, we routinely used Tn*7*R and *glmS1* and *glmS3* specific primer pairs (**Table**) for checking the locations of mini-Tn*7* insertions in subsequent experiments.)

Table. Primers used for mapping *att*Tn*7* sites in *B. ubonensis*

Primers used for mapping Tn*7* insertion sites in strain Bu278

Primer	Sequence	PCR fragment size paired with P_{Tn7L}	Reference
P_{Tn7L}	5'-ATTAGCTTACGACGCTACACC	-	Choi et al.[1]
$P_{BUGLMS1}$	5'-TCGCTCTTGAATCGACTCTTAG	379 bp	This study
$P_{BUGLMS2}$	5'-TCTTTGAAATCTACGACGAGCCGTT	467 bp	This study
$P_{BUGLMS3}$	5'-CGAAGACGTGATCCGCGAGA	285 bp	This study
$P_{BUGLMS4}$	5'-TATACGTCATGTGGCCAGTTGC	418 bp	This study

Primers used for routine mapping of Tn*7* insertion sites in *B. ubonensis*

Primer	Sequence	PCR fragment size paired with P_{Tn7R}	Reference
P_{Tn7R}	5'-CACAGCATAACTGGACTGATTTC	-	Choi et al.[1]
P3357 (*glmS1*)[2]	5'- CGAGCTGTACGTGTTCGCG	341 bp	This study
P3358 (*glmS3*)[2]	5'- GCTCTATGTGTTCGCGGATG	337 bp	This study

[1]Primer P_{Tn7R} was previously described (K.-H. Choi, J.B. Gaynor, K.G. White, C. Lopez, C.M. Bosio, R.R. Karkhoff-Schweizer, and H.P. Schweizer, Nat Methods 2:443-448, 2005).

[2]P3357 and P3358 bind within the *glmS1* and *glmS3* coding sequences

We analyzed 22 TMP^r transformants and mapped insertions 25 bp and 24 bp downstream of *glmS1* and *glmS3.* We did not observe any insertions at *glmS2* and *glmS4*, but may not have examined a sufficient number of transformants.

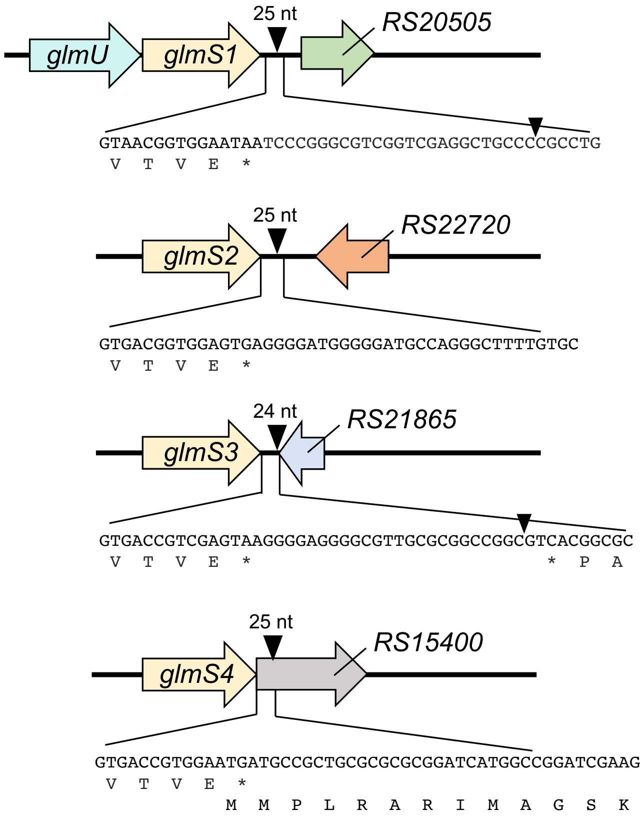

Figure 1. Mapping of Tn*7* insertion sites in *B. ubonensis* Bu278. Putative Tn*7* insertion sites (*att*Tn*7*) are indicated by arrowheads. The arrowheads above the sequence mark experimentally verified insertion sites. The *glmS1* gene is preceded by *glmU.* These genes encode two essential enzymes of the UDP-*N*-acetyl-glucosamine synthesis pathway; it follows that GlmS1 is a glucosamine-fructose-6-phosphate aminotransferase and GlmU a bifunctional enzyme that catalyzes the last two sequential reactions in the pathway. Downstream genes are labeled by RS15400, RS20505, RS21865 and RS22720 (short forms of the respective COJ66_RS annotations [www.burkholderia.com]), all of which encode hypothetical proteins.

2. Isolation of a gentamicin susceptible Bu278 mutant.

B. ubonensis Bu278 is gentamicin (GEN) resistant (Etest MIC = 48 µg/ml). To isolate a GEN susceptible Bu278 derivative for use in genetic experiments involving a GEN resistance marker, the transposon T23 library described in the main text was screened for mutants unable to grow on LB plates containing 15 µg/ml GEN. One such mutant, Bu333, was found after screening ~2,100 transposon mutants and its GEN Etest MIC was 0.19 µg/ml. The transposon insertion site in strain Bu333 was mapped by self-ligation of *Not*I-digested genomic DNA fragments, followed by rescue of plasmids containing the TMP resistance marker and the *ori* residing on T23 after transformation of *E. coli* DH5α, and Sanger sequencing of the transposon-genome junctions on rescued plasmids isolated from TMP^r transformants (K. Choi, D. DeShazer and H.P. Schweizer, Nature Protocols 1: 162-169, 2006). The transposon was inserted into codon 495 of gene COJ66_RS00470 (*amrB*) encoding the cytoplasmic membrane efflux transporter component of the AmrAB-OprA resistance nodulation cell division (RND) efflux pump known to be responsible for the intrinsic aminoglycoside resistance of several *Burkholderia* species (N.L. Podnecky, K.A. Rhodes, H.P. Schweizer, Front Microbiol 6:305, 2015, doi:10.3389/fmicb.2015.00305)(**Fig. 2**).

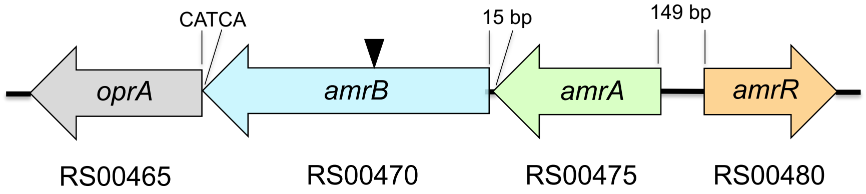

Figure 2. The *amrAB-oprA* efflux pump operon and its regulatory gene *amrR* of strain Bu278. The map shows the organization of the three genes of the *amrAB-oprA* operon that encode the three structural components of the AmrAB-OprA efflux pump (membrane fusion protein AmrA, the RND transporter AmrB and the outer membrane channel protein OprA) and the cognate TetR family transcriptional regulator AmrR. The arrowhead indicates the transposon insertion site that obliterates AmrAB-OprA function and thus results in a GEN susceptibility phenotype. The RS gene annotations are short forms of the respective COJ66_RS annotations (e.g. COJ66_RS00470 for *amrB*) (www.burkholderia.com; G.L. Winsor, B. Khaira, T. Van Rossum, R. Lo, M.D. Whiteside, F.S.L Brinkman, Bioinformatics 24:2803-2804, 2008).

3. β-lactamase expression and secretion in *E. coli*

To assess β-lactamase activities in *E. coli* the native TAT and lipoprotein signal sequences of *Bp* PenA and *Bu* PenA* and PenB, as well as the native signal sequence of *Bu* AmpC were replaced by the *E. coli* DsbA signal sequence (ssDsbA) (Schierle at al., J Bacteriol 185:5706, 2003). This was achieved by fusing the 20 amino acid DsbA signal sequence to the +9 lysine of mature *Bp* PenA, the +9 serine of *Bu* PenB, the +10 glutamate of *Bu* PenA*, as well as the +2 isoleucine of AmpC such that the signal peptides of the respective proteins was removed and the DsbA signal peptidase I cleavage site (A↓A) between amino acids 19 and 20 recreated (MJ Tsang , AA Yakhnina, and TG Bernhardt, PLoS Genet 13:e1006888, 2017). PCR fragments containing $DsbA_{ss}$ and the respective β-lactamase coding sequences without native signal sequences were assembled with pBC-SK(-) (Agilent, Santa Clara, CA) using the NEBuilder® HiFi DNA Assembly Master Mix (New England Biolabs). This placed the hybrid genes

under transcriptional control of P_{lac} and translational control of a consensus ribosome-binding site, both provided by the vector.

Functional expression in *E. coli* GBE180 was assessed by monitoring growth at 37ºC with shaking in microtiter plates containing LB medium + 25 µg/ml chloramphenicol (CHL) alone for vector maintenance or CHL + 100 µg/ml AMP for cells with plasmids encoding putative β-lactamases. The optical density at 600 nm was read at 30 min intervals in a BioTek EPOCH 2 microplate reader (Winooski, VT).

Periplasmic localization and activity were assessed by cold osmotic shock (GL Hazelbauer and S, Harayama, Cell 16:617, 1979) of 1.5 ml of a LB + CHL grown overnight culture that resulted in 0.3 ml shock fluid in 0.5 mM $MgCl_2$. β-Lactamase activity was qualitatively assessed by nitrocefin (0.5 mg/ml; Santa Cruz Biotechnology, Dallas, TX) hydrolysis mediated by 50 µl of shock fluid in 1 ml 0.1 M sodium phosphate (pH 7.0) at 37ºC (31). Alkaline phosphatase was. Used as a periplasmic marker protein. Its activity was qualitatively assessed by p-nitrophenol-phosphate (1 mM; Sigma-Aldrich) hydrolysis mediated by 20 µl of shock fluid in 1 ml 1 M Tris-HCl (pH 8.0) at 37ºC (M. Argast M and W. Boos, J Bacteriol 143:142, 1980).

Comparative β-lactamase expression in cell free extracts (CFE) was performed as previously described with minor modifications (L. Lauretti et al., Antimicrob Agents Chemother 43:1584, 1999). Briefly, *E. coli* GBE180 cells containing empty vector or plasmids expressing periplasmic $PenB_{Bu278}$ or $PenB_{MSMB2152}$ were grown at 37ºC overnight in LB + CHL medium. A 0.5 ml aliquot of each culture was added to 50 ml LB + CHL medium and grown at 37ºC to an OD_{600nm} of 0.5-1.0. Cells were harvested (12,000x*g*, 4ºC) and the pellets suspended in 1 ml cold (4ºC) phosphate-buffered saline (PBS, pH 7.4). Cells were lysed by sonication using a Sonics VibraCell sonicator (Sonics, Newton, CT) and previously described conditions (L. Lauretti et al., Antimicrob Agents Chemother 43:1584, 1999). After centrifugation at (17,000x*g*, 4ºC) the supernatant was saved as CFE. Protein was assayed using the Pierce BCA Protein Assay Kit (ThermoScientific, Rockford, IL). β-lactamase activity was determined by nitrocefin hydrolysis recorded as absorbance increase at 486 nm at 37ºC on a BioTek EPOCH 2 microplate reader. Each 0.2 ml reaction mixture in microtiter plates contained PBS (pH 7.4), 0.05 mg/ml nitrocefin and 5 µg of CFE protein.
